# Supplementary material for: Standardization transformation of C-lignin to catechol and propylene
Source: Nat Commun. 2025 Jul 7;16:6245. doi: 10.1038/s41467-025-61457-y (PMC12234704; doi:10.1038/s41467-025-61457-y)
Supplement: Supplementary file 1 — Supplementary Information [file 41467_2025_61457_MOESM1_ESM.pdf]

Standardization Transformation of C-Lignin to Catechol and Propylene

Xiaojun Shen [1,2,3,9*], Zhitong Zhao [4,9], Jialong Wen [1,3], Jian Zhang [2], Yi Ji [5,6], Guangjin Hou [5],

Yuhe Liao[7], Chaofeng Zhang[8]*, Tong-Qi Yuan [1,3*], Feng Wang [2*]

[1] State Key Laboratory of Efficient Production of Forest Resources, Beijing Forestry University, Beijing 100083, China

[2] Dalian National Laboratory for Clean Energy, Dalian Institute of Chemical Physics, Chinese Academy of Sciences, 457 Zhongshan Road, Dalian 116023, China

[3] Beijing Key Laboratory of Lignocellulosic Chemistry, Beijing Forestry University, Beijing 100083, China

[4] College of Chemical Engineering and Technology, Taiyuan University of Technology, Taiyuan 030024, China

[5] State Key Laboratory of Catalysis, Dalian Institute of Chemical Physics, Chinese Academy of Sciences, Dalian 116023, China

[6] University of Chinese Academy of Sciences, Beijing, China

[7] Guangzhou Institute of Energy Conversion, Chinese Academy of Sciences, Guangzhou 510640, Guangdong, China

[8] Jiangsu Co-Innovation Center of Efficient Processing and Utilization of Forest Resources, College of Light Industry and Food Engineering, Nanjing Forestry University, 159 LongPan Road, Nanjing 210037, China.

[9] These authors contributed equally

*Correspondence to: shenxiaojun@bjfu.ecu.cn (Xiaojun Shen), zhangchaofeng@njfu.edu.cn (Chaofeng Zhang), ytq581234@ bjfu.edu.cn (Tong-Qi Yuan), and wagnfeng@dicp.ac.cn (Feng Wang)

Table of Contents

Materials and Methods……………………………..……………………………..……..4
Materials………………………………………………………………………..……4
Methods……………………………………………………………………...….5
C-lignin preparation from Castor shell endocarp ……...………………………..…….…5
Catalyst Characterization...……………………………………………..……...…5
Computational method……………………………………..…………..………6
Process model design details…………………………………………………...….……7
Synthesis of the model compounds………………………………………...………24
4-Propenylcatechol………………………………………………………..…….24
4-(1-Propenyl)-catechol………………………………………………...……25
3',4'-Dihydroxypropiophenone………………………………..………………...…26
1-(3,4-Ethylenedioxyphenyl)propan-1-ol………………………………...………27
1-Phenylethane-1,2-diol……………………………………...……………..……28
4-Isopropenyl-1,2-dimethoxybenzene……………………………...………………29
3,4-Dimethoxycumyl alcohol………………………………………..………..30

Supplementary text
Supplementary Note 1………………………………………………...……………31
Supplementary Note 2………………………………………………………...……34
Supplementary Note 3………………………...………………………………..……40

Figures
Supplementary Fig. 1 The process model of catechol production via the biomass route……………………………………………………………………..……………..……10
Supplementary Fig. 2 The process model of catechol production via the conventional route…………………………………………………………………..…………………..…15
Supplementary Fig. 3 The system boundaries of catechol production via (a) bio-route with ball-milling and enzymatic hydrolysis; (b) bio-route with solvent extraction; (c) conventional route…………………………………………………………………………………..………18
Supplementary Fig. 4 Wide applications of catechol in the manufacturing industry……………31
Supplementary Fig. 5 Quantitative ^{13}C NMR spectrum of C-lignin (endocarp) (DMSO-*d*6) …………………………………………………………………………...………...…34
Supplementary Fig. 6 The GC spectrum of C-lignin oil with derivatisation after the reaction.……………………………………………………………………………………..…35
Supplementary Fig. 7 Mass spectra of product from C-lignin depolymerisation…………………38
Supplementary Fig. 8 The MS spectrum of (a) 1-(3,4-dihydroxyphenyl)propan-2-one and (b) 3-(3,4-dihydroxyphenyl)propanal after BSFTA derivatization, (c) The derivatization experiments of 1-(3,4-dihydroxyphenyl)propan-2-one nor 3-(3,4-dihydroxyphenyl)propanal, and (d) The potential pathway to C5 formation……………………………………………………………...39
Supplementary Fig. 9 The effect of Si/Al ratio in support of catalyst on the monomer yields…………………………………………………………………..……………..……41
Supplementary Fig. 10 Conversion of acetone into propylene………………………………...42
Supplementary Fig. 11 XRD patterns of HY_{30} and Ni/HY_{30} catalysts, a standard pattern of HY zeolite, are shown at the bottom……………………………………………………………..…44
Supplementary Fig. 12 EDS images of 0.97 wt% Ni/HY_{30}……………………...………..…45
Supplementary Fig. 13 CO-FTIR spectra of HY_{30} at different desorption time………………...46

Supplementary Fig. 14 Nitrogen adsorption-desorption isotherm of HY_{30}, HY_{30}-0.4, HY_{30}-0.8, HY_{30}-1.6 and HY_{30}-2.4zeolite……………………………..……………………………………49
Supplementary Fig. 15 (a) Nitrogen adsorption-desorption isotherm of HY_{30} and HY_{30}-C; (b) pore size distribution of HY_{30} and HY_{30}-C…………………………….……………………………………50
Supplementary Fig. 16 Hydrogenolysis-dealkylation of C-lignin obtained via BME process and solvent extraction over Ni/HY_{30} catalyst.
……………………………………………………………………………..……………52

Tables

Supplementary Table 1 The composition of castor shells in the simulation model……………….11
Supplementary Table 2 Process parameters and reaction condition of the conversion of C-lignin to catechol in the reaction unit………………………………………………………………………12
Supplementary Table 3 Block types used in Aspen plus……………….…………………….…….13
Supplementary Table 4 Components and their mass fraction of key stream in the conversion of C-lignin to catechol production………………………………………………………………………14
Supplementary Table 5 Reaction condition or key parameters of the catechol production via the petroleum route……………………………..……………………………………………….…16
Supplementary Table 6 Components and their mass fraction of keystream in the conventional phenol oxidation route for catechol production……………………………………………...…..17
Supplementary Table 7 Assumptions for the estimation of total product cost…………………..19
Supplementary Table 8 The detailed TEA results of three catechol production………………...20
Supplementary Table 9 The detailed PFE breakdown of three catechol production…………..……21
Supplementary Table 10 The detailed GHG breakdown of three catechol production.…………22
Supplementary Table 11 The detailed LCA results…..………………………………….……23
Supplementary Table 12 Chemical compositions of castor seed coats (endocarp), C-lignin (endocarp) and typical biomass…………………………...…………………………………33
Supplementary Table 13 The ICP-OES analysis of Ni/HY_{30} before and after the reaction and liquid phase………...……………………..…………………………………………………..43
Supplementary Table 14 Dealkylation of propenylcatechol into catechol using different catalysts…………………………………………………………………….…………….……47
Supplementary Table 15 Textural properties of HY zeolite…………………………….…….48
Supplementary Table 16 The chemical shift value (δ, ppm) of ^{13}C NMR spectrum of the intermediates in the reaction of C2 compound………………………………………...……..51

References…………………………………………………...……………..……………53

Supplementary Materials and Methods

Materials

All chemicals are used as purchased without further purification. Aluminum chloride (AR), magnesium turnings (AR), ammonium chloride (AR), nickel chloride dihydrate (AR), sodium acetate (AR), toluene (AR), acetone (AR), isopropyl alcohol (99.5%) and diethyl ether (AR) were purchased from Damao Chemical Reagent Factory. 1,3-Diisopropylcarbodiimide (99%), aluminum iodide (95%), chromium (III) acetylacetonate (97.5%), 1,4-benzodioxin-6-carboxaldehyde (98%), mandelic acid (99%), methylmagnesium chloride (3.0M solution in THF), bromoethane (99%), 3,4-dimethoxyacetophenone (98%) and methyl sulfoxide-d_6 [D; 99.9%, 0.03% (v/v) tetramethylsilane (TMS)] were purchased from J & K Scientific Ltd. Hydrobromic acid (47% solution), anhydrous sodium sulfate (99%), lithium aluminum hydride (97%), catechol (99%), 1, 3, 5-trioxane (99.5%), and 2-methoxy-4-propylphenol (98%) were purchased from Shanghai Aladdin Biochemical Technology Co., Ltd. Sodium chloride (AR), Sodium borohydride (AR), hydrochloric acid (38%), sulfuric acid (AR), acetic acid (AR), ethyl acetate (99.5%), petroleum ether (99%) acetonitrile (AR), anhydrous tetrahydrofuran (AR), and methanol (AR) were purchased from Sinopharm Chemical Reagent Co., Ltd. Citric acid (AR), sodium thiosulfate (AR), and dichloromethane (AR) were purchased from Xilong Scientific Co., Ltd. $HY_{5.2}$ (Si/Al=5.2), HY_{30}, HY_{60} and HY_{80} were purchased from Alfa Aesar (China) Chemical Co., LTD. ZSM-525, ZSM-550, ZSM-5100, ZSM-5200, MOR2, MOR12, MOR34, Beta2.5, Beta25, Beta40, MCM-22, MCM-41, SAPO-34 were purchased from Nankai University Catalyst Co. Ltd. Ruthenium trichloride (99.9%), and palladium chloride (99.9%) were purchased from Shenyang Research Institute of Nonferrous Metals. Chloroform-d [D; 99.9%, 0.03% (v/v) tetramethylsilane (TMS)] and methyl triphenylphosphonium bromide (98%) were purchased from Beijing Innochem Science & Technology Co., LTD. Cellic® CTec2 (100 FPU/mL) was supplied by Novozymes, Beijing, China. Potassium tert-butoxide (95%) was purchased from Shanghai Maclin Biochemical Technology Co., LTD. Dodecane (99%) was purchased from Merck LTD. Nitrogen (>99.99%), argon (>99.99%), and hydrogen (>99.99%) were provided by the Dalian Institute of Chemical Physics, Chinese Academy of Sciences. Deionised water was provided by the Institute of Chemistry, Chinese Academy of Sciences.

Methods

C-lignin preparation from Castor shell endocarp

According to our previous publication, to obtain a more representative C-lignin from Castor shell endocarp, C-lignin was prepared via two-step ball milling and enzymatic hydrolysis.[S1] The ball-milling process was performed in a planetary ball mill (FritschGMBH, Idar-Oberstein, Germany) for 300 min. To prevent overheating, 10-minute intervals were provided between every 10 min milling. 10 g ball-milled Castor shell endocarp and 5.0 mL Novozyme Celluclast (100 FPU/mL) were dispersed in acetate buffer (0.05 mM, 300 ml, pH 4.8) at 50 ^oC. Enzymatic hydrolysis was performed in a rotary shaker at 150 rpm for 48 h. After that, the mixture was separated, and the residue lignin was washed with acidic water (pH=2.0) and then freeze-dried. The dried residual lignin underwent ball-milling again for 2 h, then enzymatic hydrolysis as described above.

Catalyst Characterization

The experiment was carried out on a PANalytical X-pert PRO diffractometer with Cu-kα radiation at 40 kV and 20 mA. The 2θ range was scanned from 10 to 80 ^{o}. The TEM images of the catalyst were obtained using a JEOL JEM-2100 field emission transmission electron microscope operated at 200 kV. The morphology of the catalyst was observed by FE-SEM (JSM-7800F). The XPS spectra analysis was conducted on an ESCAB250Xi electron spectrometer, in which the binding energy was calibrated to the C 1s signal at 284.8 eV. CO-adsorption FT-IR spectra and pyridine-FTIR of the catalyst were collected on a Bruker Tensor 27 instrument. The sample was pretreated at 150 ^oC for 30 min under an argon atmosphere and cooled to room temperature. After that, background spectra were collected, and the sample was treated with the flow of CO for 30 min. Then, the flow of CO was replaced by Ar to remove the physically absorbed CO of the sample.

The desorption spectra were recorded at different exposure times to investigate the interaction between CO and catalysts. The IR cell with catalyst disk was pumped down to < 1×10^{-5} Pa, and background spectra were collected. Then, pyridine vapor was introduced to the IR cell for adsorption for 30 min. The spectra were recorded after the system had been vacuumed for 120 min to remove the physically absorbed pyridine. H_2-TPR was conducted using a catalyst characterization system (Autochem 2920, Micromeritics) with a TCD detector. N_2 adsorption−desorption isotherms were acquired on an ASAP 2460 apparatus from Micromeritics. Before measurement, the sample was degassed at 300 °C under a vacuum for 24 h.

Computational method

We have employed the Vienna Ab Initio Package (VASP)[S2, S3] to perform all the density functional theory (DFT) calculations within the generalized gradient approximation (GGA) using the PBE[S4] formulation. We have chosen the projected augmented wave (PAW) potentials [S5, S6] to describe the ionic cores and take valence electrons into account using a plane wave basis set with a kinetic energy cutoff of 400 eV. Partial occupancies of the Kohn−Sham orbitals were allowed using the Gaussian smearing method and a width of 0.05 eV. The electronic energy was considered self-consistent when the energy change was smaller than 10^{-5} eV. Geometry optimization was considered convergent when the force change was smaller than 0.02 eV/Å. Grimme's DFT-D3 methodology [S7] was used to describe the dispersion interactions. An HY zeolite cluster in a cubic box of 20 Å in side length was used for all the calculations. During structural optimization, the gamma point in the Brillouin zone was used for k-point sampling, and all atoms were allowed to relax.

Process model design details

The process design is divided into three major sections: feedstock pretreatment, conversion of lignin into catechol via tandem reaction, and separation and purification of catechol. Each section can consist of several steps. The feedstock pretreatment is split into biomass broken and hydrolysis, while conversion of lignin into catechol includes divided into lignin to 4-propenyl catechol and 4-propenyl catechol to catechol. The separation and purification process includes residual solid removal, gas-liquid separation, catechol purification, hydrogen recovery, and waste heat boiler, as shown in Supplementary Fig.1.

Specifically, castor shells were used as the feedstock, and their composition was simplified based on Supplementary Table 1. The feedstock primarily consists of cellulose, hemicelluloses and lignin, with lignin serving as the key substrate for catalytic conversion, while the other components require removal. The pretreatment process was designed to efficiently separate lignin while converting carbohydrates into fermentable sugars. To achieve this, two different methods were employed for lignin extraction: ball-milling enzymatic hydrolysis (BME)[S1] and solvent extraction with aqueous ammonia[S8]. In the BME method, ball-milling was used to break the crosslinking structure between cellulose and lignin, facilitating lignin separation. The remaining carbohydrates were then hydrolyzed enzymatically using cellulase and sodium acetate buffer for 48 hours, achieving a 90% conversion of cellulose into glucose and xylose. Due to the higher solubility of glucose in water compared to lignin, water was used to effectively separate the sugars. The biomass-water mixture was filtered to remove glucose, leaving behind a solid residue composed of lignin with approximately 8.52% remaining cellulose, which was subsequently used as feedstock for catechol production. In the solvent extraction method [S8], castor seed coat endocarp was treated with a 15% ammonia solution at a solid-to-liquid ratio of 16:1, heated to 80 °C for 24

h in a glass reactor, then cooled and filtered. Ammonia was removed by vacuum distillation, and lignin was precipitated by slowly adding a 10% dilute sulfuric acid solution until the pH reached 4. The solution was then concentrated by removing half of the water, followed by cooling, filtration, and drying to obtain C-lignin with an extraction yield of 60.1%. The remaining carbohydrate-rich residue from this process was further subjected to enzymatic hydrolysis using cellulase and sodium acetate buffer for 48 h, leading to a 94% conversion of the remaining carbohydrates into glucose and xylose. This approach ensures efficient biomass utilization by separating lignin for catalytic conversion while simultaneously generating fermentable sugars from cellulose and hemicelluloses. Both methods enabled effective lignin recovery while preserving its structure for subsequent catalytic conversion. The extracted lignin fractions were used as substrates for catechol production.

The conversion from pretreated feedstock into catechol is divided into two steps: lignin to 4-propenyl catechol and 4-propenyl catechol to catechol. The first reaction occurs when the lignin enters the reactor with a catalyst and a mixture of solvent (water and methanol with a molar ratio of 4:1) at 200 °C and 3 MPa. The products, which include water and 4-propenyl catechol, are produced after 12 h. The details of the key reaction, conversion rate, and process parameters for this step are depicted in Supplementary Table 2. The product mixture then directly occurs the next reaction with a 3 MPa hydrogen atmosphere is introduced and maintained, and the temperature is in parallel with the first step. After the second reaction, the raw products, which include catechol and propylene, are shown in Supplementary Table 4, with mass contents of 0.73% and 5.59%, respectively. This mixture then undergoes a separation process, where the gaseous components are transferred via a cyclone separator. The cyclone separator is used to separate the solid residue from the gas-phase mixture. The separated solids are sent to a furnace for combustion, while the gas-

phase components (including products and solvents) proceed to further purification stages to generate the qualified products.

The separation and purification include solid removal, gas-liquid separation, catechol purification, hydrogen recovery and recycling, and waste combustion for heat generation. At first, unreactive lignin and cellulose are removed through a cyclone separator to reduce the risk of pipe blockage, followed by a gas-liquid separator at 40 °C. The liquid flow contains water, methanol, catechol and chroman-6,7-diol, while the gas flow consists of hydrogen and propylene. The former is heated at about 90 °C to remove most of the water and methanol in the flash separator, and further enters the column to limit water content 0.001%. The treated liquid flow contains 96.97% of catechol and 3.03% of chroman-6,7-diol. The purification column is arranged subsequently, resulting in a purity of 99.8% of catechol in the final product. On the other hand, the gas flow, including hydrogen, propylene and less water and methanol, is passed by the cooler, heat exchanger and expansion valve, resulting in a low temperature of -70 °C. The propylene and other components are liquified and removed from the gas flow. The mass fraction of propylene is 42.81%, which is hard to sell as an acceptance. Thus, the separated propylene is sent to the boiler to provide heat for the whole plant, while hydrogen is recovered to be used as feedstock.

As a comparison, catechol production via a petroleum route is also considered. Three feedstocks, phenol, hydrogen peroxide and water, are mixed with a molar ratio of 1:1:55.5, then heated and entered into the reactor. The reaction occurs at 60 °C and 1 atm, including conversion from phenol and H_2O_2 into catechol and water, as well as co-reaction *p*-hydroquinone generation. The details of the key reaction, conversion rate, and process parameters for this step are depicted in Supplementary Table 5. The product flow includes catechol, *p*-hydroquinone, water, unreactive phenol and H_2O_2. Light fraction, water, phenol and H_2O_2 could be removed in sequence in

distillation columns. Note that the mass fraction of water approaches 90% in the raw product flow. Thus, a multiple-effect evaporation method is arranged. The overhead distillate contains water, slight phenol and H_2O_2, which is recycled together with feedstock. The bottom flow from the last flash of the multiple-effect evaporation is sent to the light column, where a mass fraction of the light fraction is limited to 0.01%. At last, the purification column is arranged to separate *p*-hydroquinone, as shown in Supplementary Fig. 2 and Supplementary Table 6.

The overall process design is simulated by Aspen Plus to estimate detailed material and energy flow according to the process parameters mentioned above. The assumed mass of feedstock is 10 metric tons per hour (t/h). The product mass will be 2.2 t/h. The process flow diagram is shown in Supplementary Figs. 1 and 2. The simulation blocks of unit equipment are depicted in Supplementary Table 3, and the parameters of key units can be found in Supplementary Tables 2 and 4. The components of the key stream are shown in Supplementary Tables 3 and 5.

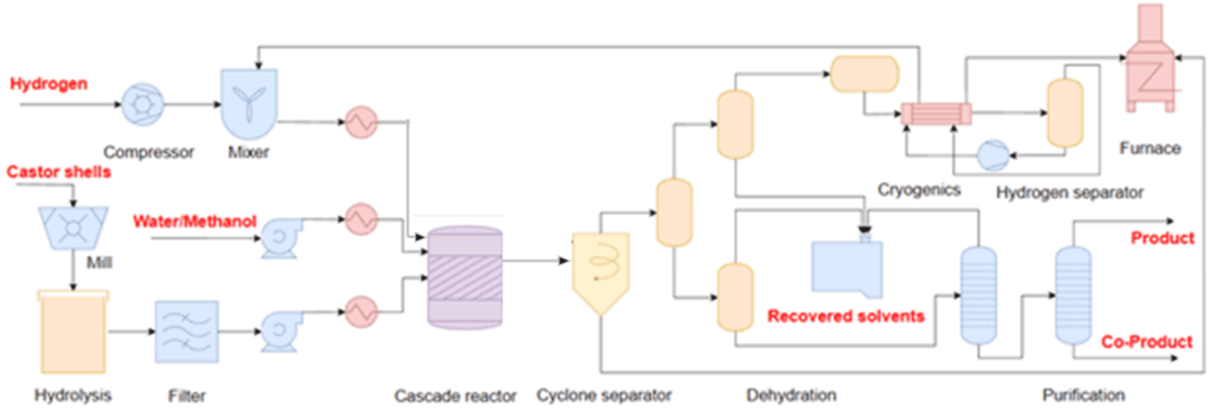

Supplementary Fig. 1 The process model of catechol production via the biomass route

Supplementary Table 1 The composition of castor shells in the simulation model

Component	Mass content	According to the component in Aspen
Cellulose	16.11%	Cellulose
Hemicelluloses	10.13%	Cellulose
Lignin	58.49%	Lignin
Water	10.27%	Water
Inorganic salt	0.05%	SiO_2

Supplementary Table 2 Process parameters and reaction condition of the conversion of C-lignin to catechol in the reaction unit

Reaction condition		Reaction product	
Temperature	200 °C	Product	Chroman-6,7-diol
Pressure	3 MPa	Coproduct	Water
Solvent	Water/methanol		
Substrate	Lignin		
Ratio of solvent to substrate	10		
Conversion rate	53.0%		

Supplementary Table 3 Block types used in Aspen plus

Process step	Type
Ball mill or solvent extraction	Reference
Enzyme hydrolysis reactor	RStoic (Stoichiometric reactor)
Lignin to 4-propenyl catechol reactor.	RStoic (Stoichiometric reactor)
4-Propenyl catechol to catechol reactor	RStoic (Stoichiometric reactor)
Flash drum	Flash2 (Two-outlet flash)
Rectifying column	RadFrac (Rigorous fractionation)
Solid-liquid separator	Sep2 (Two-outlet component separator)
Heater and cooler	Heater (Heater/cooler)
Gas compressor	MCompr (Multistage compressor/turbine)

Supplementary Table 4 Components and their mass fraction of key stream in the conversion of C-lignin to catechol production

Flowrate	Castor shells	Solvent	Hydrogen	Cycle hydrogen	Raw product	Coproduct	Product
Mass/kg	1000	6608	11.6	2169	2900	6.5	210
Mass distribution (%)							
Cellulose	26.24				0.12		
Lignin	58.49				0.92		
Water	10.27	83.40			19.40		0.02
Methanol		16.60			3.57		
Hydrogen			100	92.90	69.48		
Catechol					0.73	3.25	99.86
Propylene				7.10	5.59		
Chroman-6,7-diol					0.02	96.75	0.12
Ash	5.00				0.17		

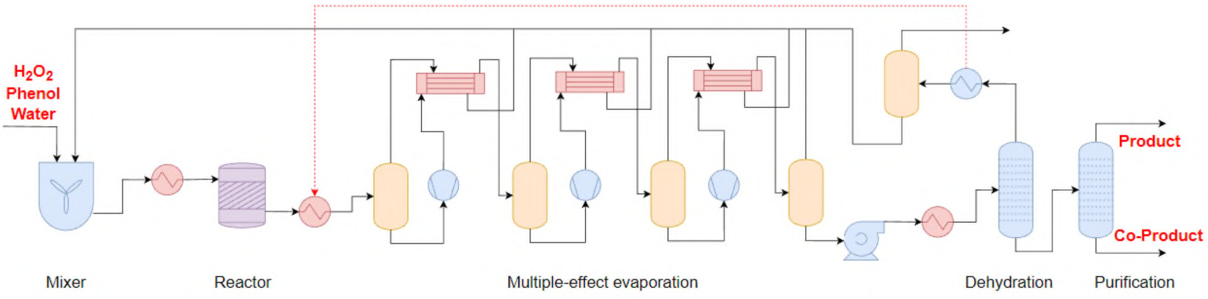

Supplementary Fig. 2 The process model of catechol production via the conventional route

Supplementary Table 5 Reaction condition or key parameters of the catechol production via the petroleum route

Reaction condition	Design
Temperature	60 °C
Pressure	1 atm
% Conversion of phenol: H_2O_2 + Phenol → Catechol + H_2O	13.5%
% Conversion of phenol: H_2O_2 + Phenol →*p*-hydroquinone + H_2O	14.1%

Supplementary Table 6 Components and their mass fraction of keystream in the conventional phenol oxidation route for catechol production

Flowrate	Feedstock	Cycle feedstock	Raw product	Product	Coproduct
Mass/kg	8643	216997	225640	2976	3108
Mass distribution (%)					
H_2O_2	22.05	2.26	2.18		
Phenol	62.19	6.20	6.04	0.18	
H_2O	15.76	91.53	89.06		
Catechol		0.02	1.34	99.80	0.10
p-Hydroquinone		trace	1.38	0.16	99.90

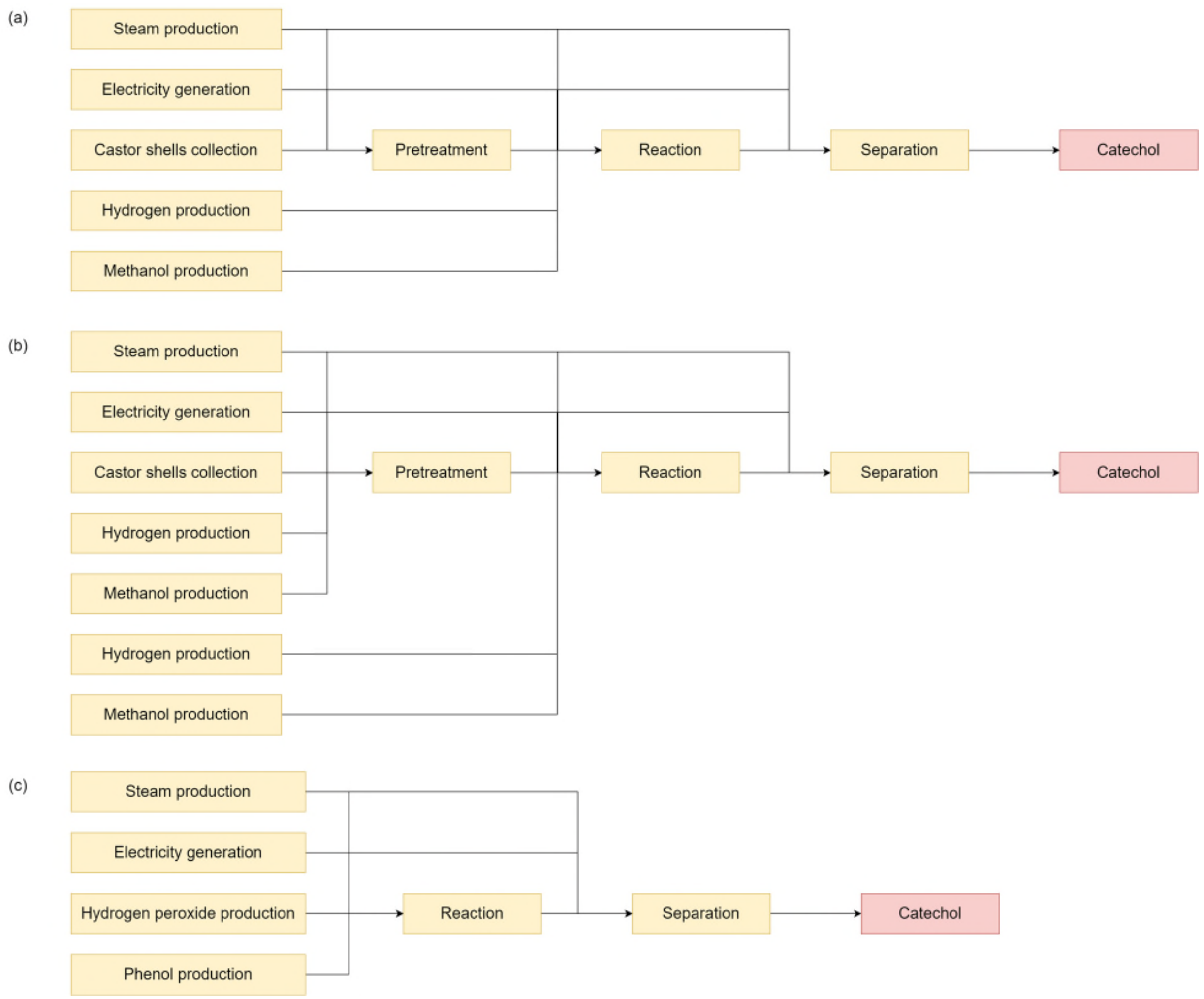

Supplementary Fig. 3 The system boundaries of catechol production via (a) bio-route with ball-milling and enzymatic hydrolysis; (b) bio-route with solvent extraction; (c) conventional route

Supplementary Table 7 Assumptions for the estimation of total product cost

Component		Base
**Total capital investment**		
Inside battery limits	(1.1)	Installed cost of all equipment
The installed cost of other equipment		Aspen Process Economic Analyzer
Outside battery limits	(1.2)	20% of (1.1)
Indirect costs	(1.3)	60% of ((1.1) + (1.2))
Fixed capital investment	(1.4)	(1.1)+(1.2)+(1.3)
Working capital	(1.5)	5% of (1.4)
**Total production cost**		
Raw materials cost	(2.1)	Castor shells: 100 CNY/t<br>Hydrogen: 10000 CNY/t<br>Methanol: 2000 CNY/t<br>Cellulase: 2200 CNY/L<br>Process water: 15 CNY/t<br>Hydrogen peroxide: 1200 CNY/t<br>Phenol: 7000 CNY/t
Consumables cost	(2.2)	Ammonia: 3600 CNY/t<br>Sulphuric acid: 400 CNY/t
Utilities cost	(2.3)	Cooling water: 3 CNY/t<br>Electricity: 0.65 CNY/kW·h<br>Steam: 200 CNY/t
Operating & maintenance cost	(2.4)	
Operating labours	(2.4.1)	60 operators,<br>100,000 CNY/operator/year
Direct supervisory & clerical labour	(2.4.2)	20% of (2.4.1)
Maintenance and repairs	(2.4.3)	2% of (1.4)
Operating supplies	(2.4.4)	0.8% of (1.4)
Laboratory charge	(2.4.5)	15% of (2.4.1)
Depreciation	(2.5)	Life period 20y, salvage value 4%
Plant overhead cost	(2.6)	60% of ((2.4.1) + (2.4.2) + (2.4.3))
Administrative cost	(2.7)	2% of product cost
Distribution and selling cost	(2.8)	2% of product cost

Supplementary Table 8 The detailed TEA results of three catechol production (CNY/t)

	Petro-catechol	Bio-catechol via BME	Bio-catechol via solvent extraction
Raw materials	6793.74	4187.62	4187.62
Consumables			3628.99
Utilities	2650.61	17701.59	2279.00
By-Product		-2061.34	-2061.34
Operating & maintenance cost			
Operating labours	12.00	12.00	12.00
Direct supervisory & clerical labor	2.40	2.40	2.40
Maintenance and repairs	52.51	728.29	728.29
Operating supplies	21.00	291.32	291.32
Laboratory charge	1.80	1.80	1.80
Depreciation	126.03	1747.89	1747.89
Plant overhead cost	8.64	8.64	8.64
Administrative cost	201.43	471.25	225.55
Distribution and selling cost	201.43	471.25	225.55
Total production cost	10071.60	23562.72	11277.72

Supplementary Table 9 The detailed PFE breakdown of three catechol production

	Petro-catechol	Bio-derived catechol via BME	Bio-catechol via solvent extraction
Hydrogen		0.05%	12.69%
Methanol		0.00%	0.14%
Ammonia			59.08%
Electricity	0.00%	99.90%	14.01%
Steam	36.32%	0.03%	8.31%
H_2SO_4			0.21%
Castor shells		0.02%	5.57%
H_2O_2	4.95%		
Phenol	58.72%		

Supplementary Table 10 The detailed GHG breakdown of three catechol production

	Petro-catechol	Bio-derived catechol via BME	Bio-catechol via solvent extraction
Hydrogen		2.56%	10.62%
Methanol		1.74%	7.21%
Ammonia			51.11%
Electricity	0.00%	92.98%	19.48%
Steam	53.30%	1.71%	7.09%
H_2SO_4			0.26%
Castor shells		1.02%	4.22%
H_2O_2	7.72%		
Phenol	38.58%		

Supplementary Table 11 The detailed LCA results

Petro-catechol			Bio-catechol via solvent extraction			Bio-catechol via BME process		
Feedstock								
H_2O_2	0.64	t	H_2	0.06	t	H_2	0.06	t
Phenol	1.87	t	Castor shells	4.75	t	Castor shells	4.75	t
Water	0.45	t	Water	28.52	t	Water	28.52	t
			Methanol	0.12	t	Methanol	0.12	t
			$NH_3 \cdot H_2O$	2.01	t			
			H_2SO_4	0.29	t			
Product								
Catechol	1	t	Catechol	1	t	Catechol	1	t
Hydroquinone	1.04	t	Chroman-6,7-diol	0.03	t	Chroman-6,7-diol	0.03	t
Utilities								
Cooling water	464.88	t	Cooling water	30.38	t	Cooling water	30.38	t
Electricity	0.12	kWh	Electricity	1236.97	kWh	Electricity	24758.62	kWh
High steam	3.30	t	Steam	4137.31	MJ	Steam	4137.31	MJ
Low steam	23.09	t						

Synthesis of the model compounds

4-Propenylcatechol

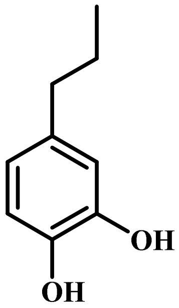

Hydrobromic acid (47% solution, 0.4 mL) was added into 2-methoxy-4-propylphenol (100 mg, 0.6 mmol). The reaction mixture was stirred at 120 °C for 20 h. after the reaction, diethyl ether (5 mL) was added to the stirred water layer at room temperature. The water layer was then successively washed with diethyl ether (5 mL×3) and saturated brine (20 mL) and dried with anhydrous Na_2SO_4. The solvent was removed via a rotary evaporator, and the product was purified by column chromatography (petroleum ether: ethyl acetate=2:1) to obtain 2-propylbenzene-1,2-diol. ^{1}H NMR (700 MHZ, DMSO-d_6): δ 8.65 (s, 1H), 8.55 (s, 1H), 6.61 (d, J = 7.9 Hz, 1H), 6.54 (d, J = 2.1 Hz, 1H), 6.39 (dd, J = 8.0, 2.0 Hz, 1H), 2.35 (t, J = 7.6 Hz, 2H), 1.49 (q, J = 7.5 Hz, 2H), 0.84 (t, J = 7.3 Hz, 3H). ^{13}C NMR (176 MHZ, DMSO-d_6): δ 144.92, 143.06, 132.93, 118.87, 115.70, 115.36, 36.72, 24.33, 13.65.

4-(1-Propenyl)-catechol

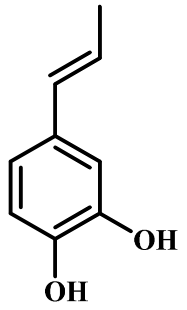

1,3-Diisopropylcarbodiimide(DIC) (46.1 mg, 0.37 mmol) and aluminum iodide (AlI_3, 273 mg. 0.67 mmol) were added into the solution of isoeugenol (100 mg, 0.61 mmol) in acetonitrile (15 mL) under argon atmosphere. The reaction mixture was stirred at 80 ^oC for 18 h. after the reaction, the mixture was quenched with citric acid and extracted with ethyl acetate three times. The organic phase was washed with saturated sodium thiosulfate solution and brine and was dried with Na_2SO_4. The solvent was removed via a rotary evaporator, and the product was purified by column chromatography (petroleum ether: ethyl acetate=4:1) to obtain 4-(1-propenyl)-catechol. ^1H NMR (400 MHZ, DMSO-d_6): δ 8.83 (s, 2H), 6.76 (d, J = 2.0 Hz, 1H), 6.65 (d, J = 8.1 Hz, 1H), 6.61 (dd, J = 8.2, 2.0 Hz, 1H), 6.21 (d, J = 15.8 Hz, 1H), 6.04 – 5.86 (m, 1H), 1.78 (d, J = 6.6 Hz, 3H). ^{13}C NMR (176 MHZ, DMSO-d_6): δ 145.68, 145.05, 131.40, 129.57, 122.00, 117.79, 116.01, 113.15, 18.62.

3',4'-Dihydroxypropiophenone

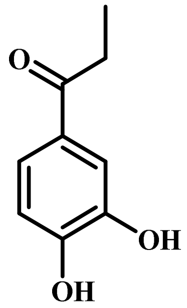

Aluminum chloride (80.0 mg, 0.6 mmol) and catechol (72.7 mg, 0.66 mmol) were added to the anhydrous dichloromethane (10 mL). The solution of propionyl chloride (92.5 mg, 0.6 mmol) in dichloromethane (5 mL) was added dropwise for over an hour, and stirring was continued for 4 h at room temperature. After the reaction, the mixture was poured onto a 15 mL ice-cold solution of concentrate (3 mmol/L) and extracted with ethyl acetate (10mL×3) and saturated brine (20 mL) and dried with anhydrous Na_2SO_4. The solvent was removed via a rotary evaporator, and the product was purified by column chromatography (petroleum ether: ethyl acetate=10:1) to obtain 3',4'-dihydroxypropiophenone. ^{1}H NMR (700 MHz, DMSO-d_6): δ 9.80 (s, 1H), 9.32 (s, 1H), 7.51 – 7.29 (m, 2H), 6.83 (d, J = 8.0 Hz, 1H), 2.89 (q, J = 7.2 Hz, 2H), 1.17 – 0.92 (m, 3H). ^{13}C NMR (176 MHZ, DMSO-d_6): δ 198.68, 150.46, 145.19, 128.67, 121.10, 115.09, 114.80, 30.57, 8.53.

1-(3,4-Ethylenedioxyphenyl)propan-1-ol

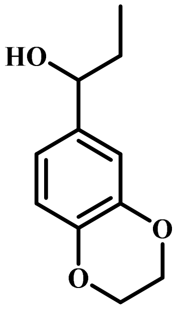

1,4-Benzodioxin-6-carboxaldehyde (82 mg, 0.5 mmol) was added to the solution of Grignard reagent that was freshly prepared from bromoethane (80.0 mg, 0.75 mmol) and magnesium turnings (20 mg, 0.75 mmol) in anhydrous tetrahydrofuran (5 mL) at 0 °C. The reaction mixture was stirred at room temperature for 1 h. After the reaction, the mixture was quenched with cold water (0.5 mL) and acidified with saturated ammonium chloride solution (5 mL). Ethyl acetate (15 mL) was added to the organic layer at room temperature. The mixture was extracted with ethyl acetate three times. The organic phase was washed with saturated deionised water and brine and dried with Na_2SO_4. The solvent was removed via a rotary evaporator, and the product was purified by column chromatography to obtain 1-(3,4-ethylenedioxyphenyl)propan-1-ol. ^{1}H NMR (400 MHZ, DMSO-d_6): δ 6.87 – 6.66 (m, 3H), 4.97 (d, J = 4.4 Hz, 1H), 4.31 (td, J = 6.3, 4.4 Hz, 1H), 4.15 (s, 4H), 1.66 – 1.46 (m, 2H), 0.80 (t, J = 7.4 Hz, 3H). ^{13}C NMR (101 MHZ, DMSO-d_6): δ 143.32, 142.41, 139.84, 119.09, 116.79, 114.92, 73.62, 64.51, 64.44, 32.45, 10.60.

1-Phenylethane-1,2-diol

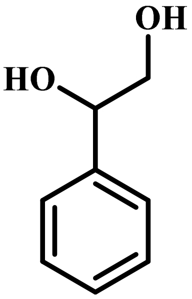

Mandelic acid (1.0 g, 6.3 mmol) was added to anhydrous tetrahydrofuran (20 mL), and a solution of lithium aluminum hydride (2 M in anhydrous tetrahydrofuran, 20 mL, 36 mmol) was added dropwise over a period of an hour. The mixture was heated to reflux and stirred for 16 h. Then, the mixture was cooled to 0 ^{o}C and quenched via dropwise adding water (5 mL) and KOH (2 M). The precipitate was dissolved by adding HCl (1M) and extracted with ethyl acetate three times. The organic phase was washed with saturated deionised water and brine and dried with Na_2SO_4. The solvent was removed via a rotary evaporator, and the product was without further purification. ^{1}H NMR (400 MHZ, DMSO-d_6): δ 7.41 – 7.26 (m, 4H), 7.26 – 7.18 (m, 1H), 5.21 (d, J = 4.2 Hz, 1H), 4.70 (t, J = 5.8 Hz, 1H), 4.53 (td, J = 6.0, 4.2 Hz, 1H), 3.42 (t, J = 5.9 Hz, 2H). ^{13}C NMR (101 MHZ, DMSO-d_6): δ 143.90, 128.27, 127.23, 126.73, 74.30, 67.97.

4-Isopropenyl-1,2-dimethoxybenzene

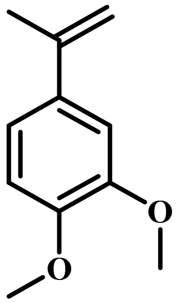

Potassium tert-butoxide (67.3 mg, 0.6 mmol) was added to the solution of methyl triphenylphosphonium bromide (214.3 mg, 0.6 mmol) in anhydrous tetrahydrofuran (5 mL) under an argon atmosphere, and stirred at room temperature for 1 h to obtain yellow suspension. Then, a solution of 3, 4-dimethoxy acetophenone (90.0 mg, 0.5 mmol) in anhydrous tetrahydrofuran was added dropwise over an hour, and the mixture was further stirred at room temperature overnight. After the reaction, water and dichloromethane were added to the mixture, and the aqueous phase was extracted with dichloromethane (10 mL×3. The combined organic phases were washed with saturated brine (20 mL) and dried with anhydrous Na_2SO_4. The solvent was removed via a rotary evaporator, and the product was purified by column chromatography to obtain 4-Isopropenyl-1,2-dimethoxybenzene. ^{1}H NMR (700 MHZ, Chloroform-d): δ 7.06 – 6.99 (m, 2H), 6.83 (d, J = 8.8 Hz, 1H), 5.38 – 5.21 (m, 1H), 5.10 – 4.95 (m, 1H), 3.90 (d, J = 14.7 Hz, 6H), 2.18 – 2.09 (m, 3H). ^{13}C NMR (176 MHz, Chloroform-d) δ 148.66, 142.81, 134.19, 118.02, 110.99, 110.76, 108.82, 55.85, 21.96.

3,4-Dimethoxycumyl alcohol

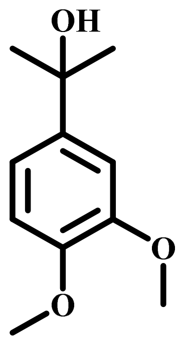

Methylmagnesium chloride solution (3.0 M in anhydrous tetrahydrofuran, 10 mL) was added to the solution of 3, 4-dimethoxy acetophenone (1.4 g, 10 mmol) in anhydrous tetrahydrofuran (20 mL) at 0 °C. The reaction mixture was stirred at room temperature for 1 h. After the reaction, the mixture was quenched with cold water (1 mL) and acidified with saturated ammonium chloride solution (20 mL). Ethyl acetate (50 mL) was added to the stirred organic layer at room temperature. The organic layer was then successively washed with deionised water (50 mL×3) and saturated brine (50 mL) and dried with anhydrous Na_2SO_4. After the concentration in vacuum rotavap, 3,4-dimethoxycumyl alcohol was obtained. ^{1}H NMR (700 MHz, Chloroform-*d*) δ 7.09 (d, *J* = 2.1 Hz, 1H), 6.98 (dd, *J* = 8.3, 2.1 Hz, 1H), 6.82 (d, *J* = 8.3 Hz, 1H), 3.88 (d, *J* = 20.4 Hz, 6H), 1.88 – 1.72 (m, 1H), 1.58 (s, 6H).^{13}C NMR (176 MHz, Chloroform-*d*) δ 148.65, 147.73, 141.98, 116.36, 110.70, 108.30, 76.88, 72.33, 55.91, 31.80.

Supplementary Note 1

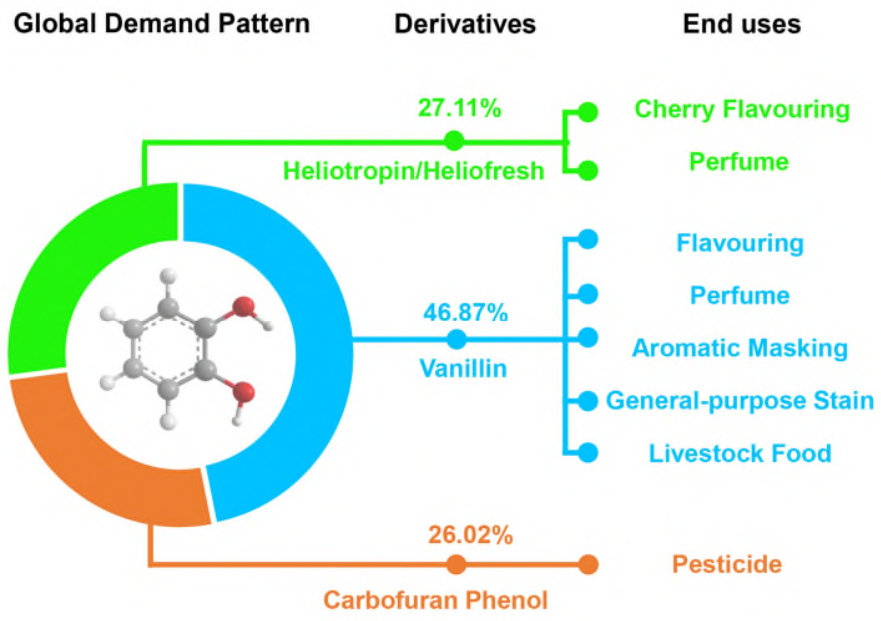

Supplementary Fig. 4 Wide applications of catechol in the manufacturing industry.

Traditional Route for catechol production from fossil resources

Catechol is an important commodity chemical in the industry with more than 44 thousand metric tons globally in 2021 [S10]. The market value of global catechol was at 300.7 million dollars in 2021 and will increase with a compound annual growth rate of 8.44% from 2020 to 2027 [S10]. Especially in the chemical industry, catechol is an important raw material for manufacturing various industrial chemical intermediates, such as vanillin, carbofuran phenol, heliotropin and heliofresh, which is extensively employed in many sectors (Supplementary Fig. 4). Furthermore, the global catechol demand is far more than the global production. Therefore, it is urgent to seek a solution for increasing the production of catechol. Traditionally, catechol is dominantly produced via dehydrogenation of 1,2-cyclohexanediol and hydroxylation of phenol. The above routes of catechol relied heavily on fossil resources and were limited by the high cost,

complicated conditions, and high energy consumption. Hence, the more sustainable and efficient synthetic route of catechol, such as using renewable and non-edible lignocellulose as the feedstock to selectively produce catechol, has great potential, which can decouple chemicals from fossil resources and decrease CO_2 emissions. Annually, plentiful lignocellulose is harvested worldwide, but most of them can't be effectively utilised.

Supplementary Table 12 Chemical compositions of castor seed coats (endocarp), C-lignin (endocarp) and typical biomass [a]

Sample	Yield (wt%)	Chemical composition （wt%）		
		Lignin	Cellulose	Hemicelluloses
Typical biomass	-	16-30	40-50	25-35
Castor shell	-	58.32	23.45	16.81
C-lignin[b]	57.1[d]	81.42	3.35	8.47
C-lignin[c]	60.1[d]	75.41	4.47	7.56

[a] The chemical compositions (wt%, w/w) of all the samples were determined according to the NREL standard analytical method (NREL/TP-510–42618)[S11].

[b] C-lignin obtained via the BME process

[c] C-lignin obtained via solvent extraction

[d] based on the content of the lignin in castor shell

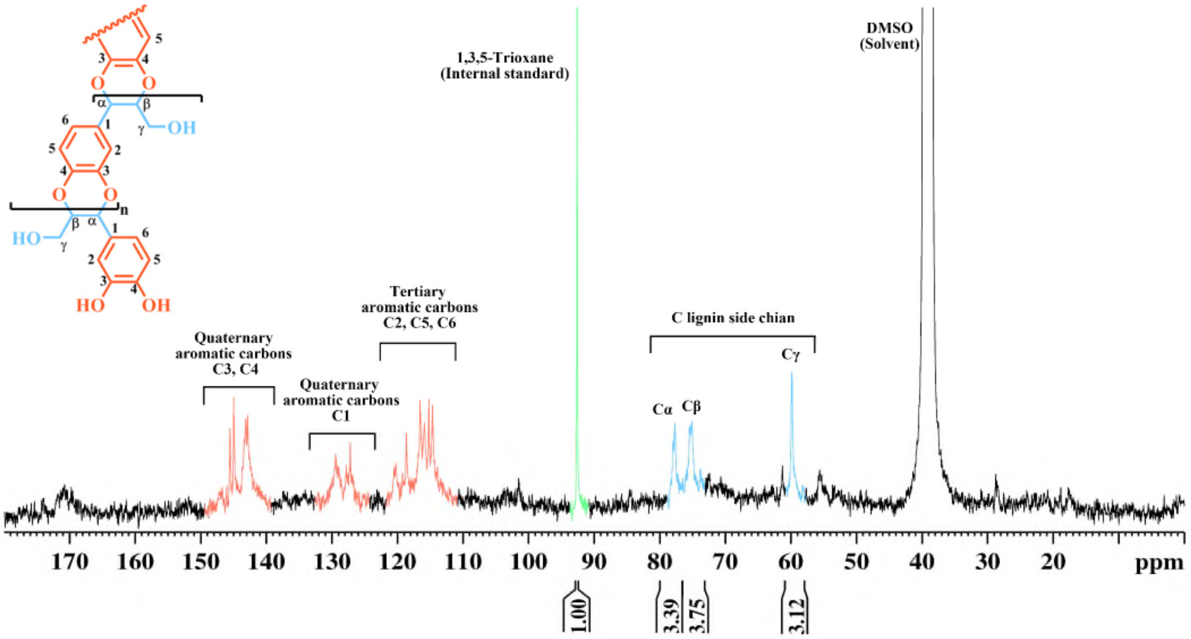

Supplementary Fig. 5 Quantitative ^{13}C NMR spectrum of C-lignin (endocarp) (DMSO-d_6)

Supplementary Note 2. The quantification of caffeyl alcohol units in isolated C-lignin was performed according to blow formulas [S12]**,** and C_β was chosen as the calculation refer.

The molar concentration of caffeyl alcohol in C-lignin (Y_{CA} (mol/%)):

$$Y_{CA}=\frac{A_{C\beta}\times n_{IS}}{\frac{A_{IS}}{3}\times W_{lignin}}$$

n_{IS} (mol) represent the mole number of internal standard (IS) (1,3,5-trioxane);

$A_{C\beta}$ represent the peak integral of C_β in the quantitative ^{13}C NMR spectrum;

A_{IS} represent the peak integral of IS in the quantitative ^{13}C NMR spectrum;

W_{lignin} (g) represent the mass of lignin;

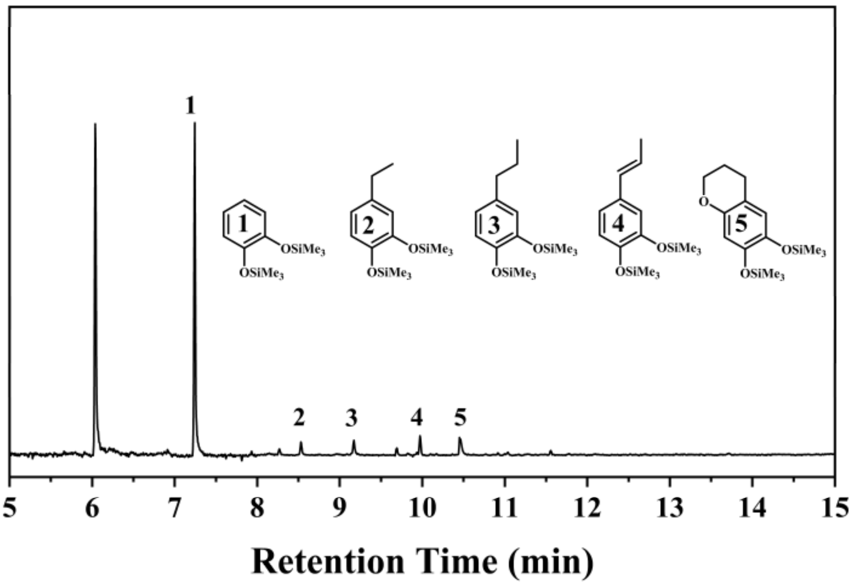

Supplementary Fig. 6 The GC spectrum of C-lignin oil with derivatisation after the reaction. Reaction condition: 50 mg C-lignin, 100 mg 0.97 wt% Ni/HY_{30}, 25 μL dodecane, 5 mL methanol/water mixture (1:4), 3 MPa H_2, 200 ^oC, 12 h.

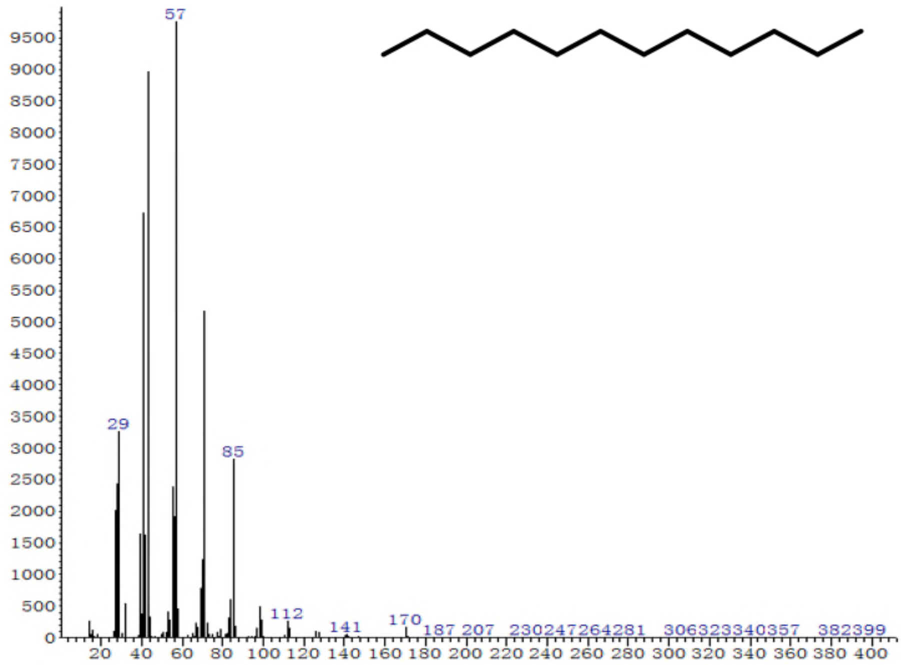
57
29
85
112
141
170
187 207 230247264281 306323340357 382399

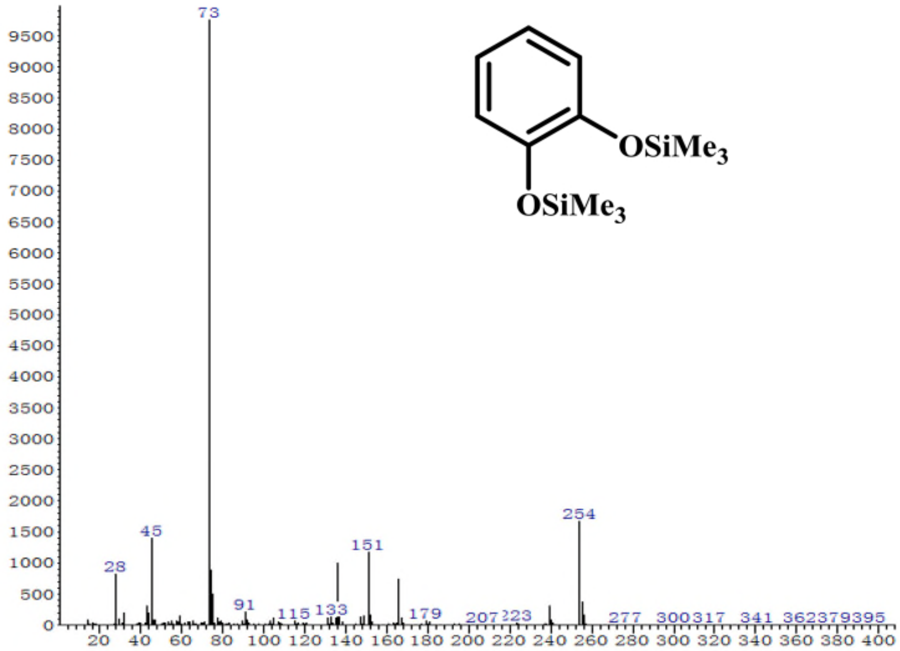
73
OSiMe3
OSiMe3
254
45
151
28
91
115 133
179
277 300317 341 362379395

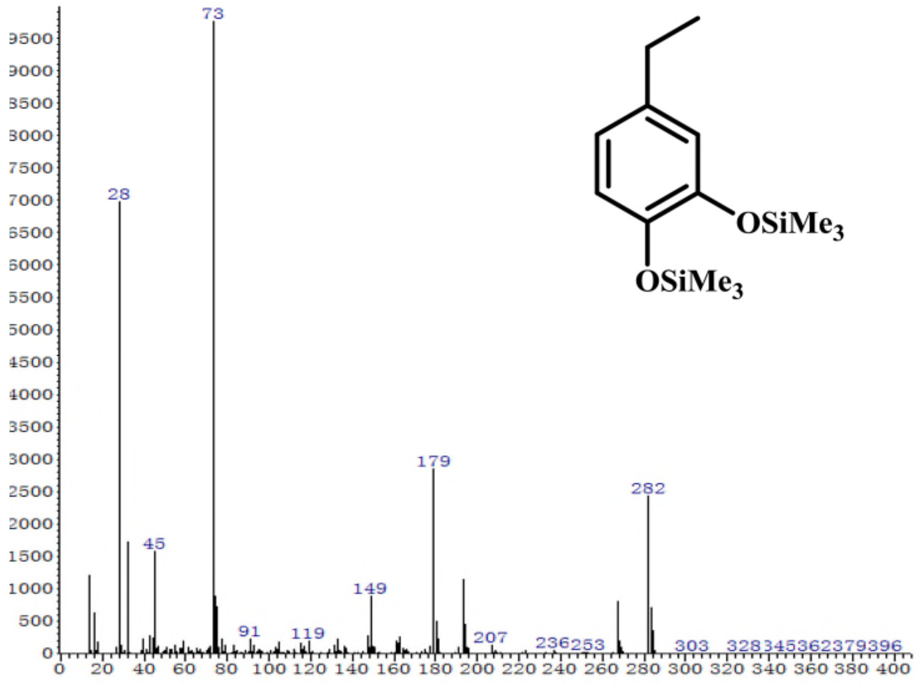
OSiMe3
OSiMe3

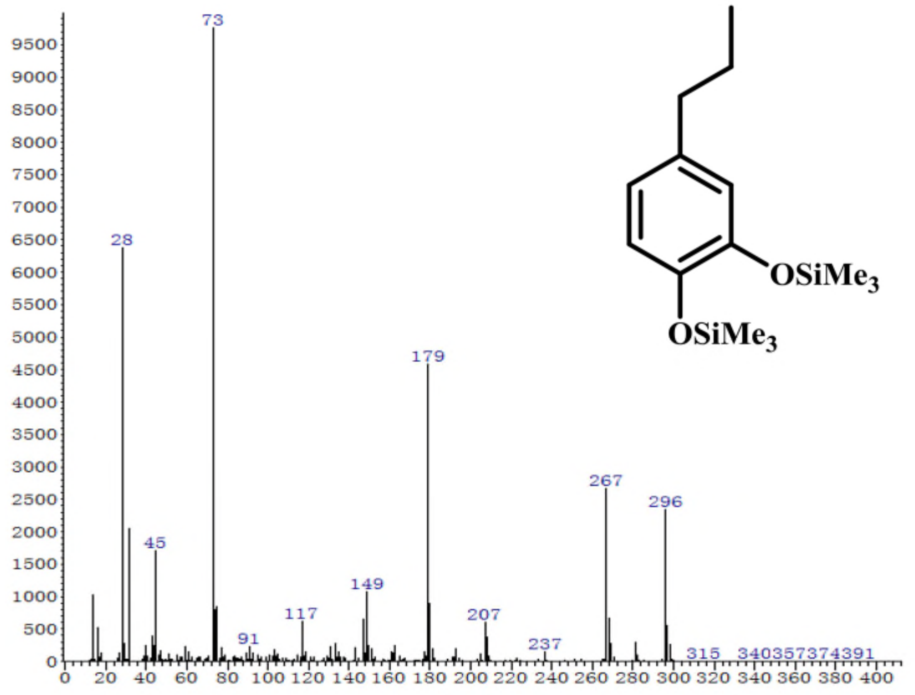
OSiMe3
OSiMe3

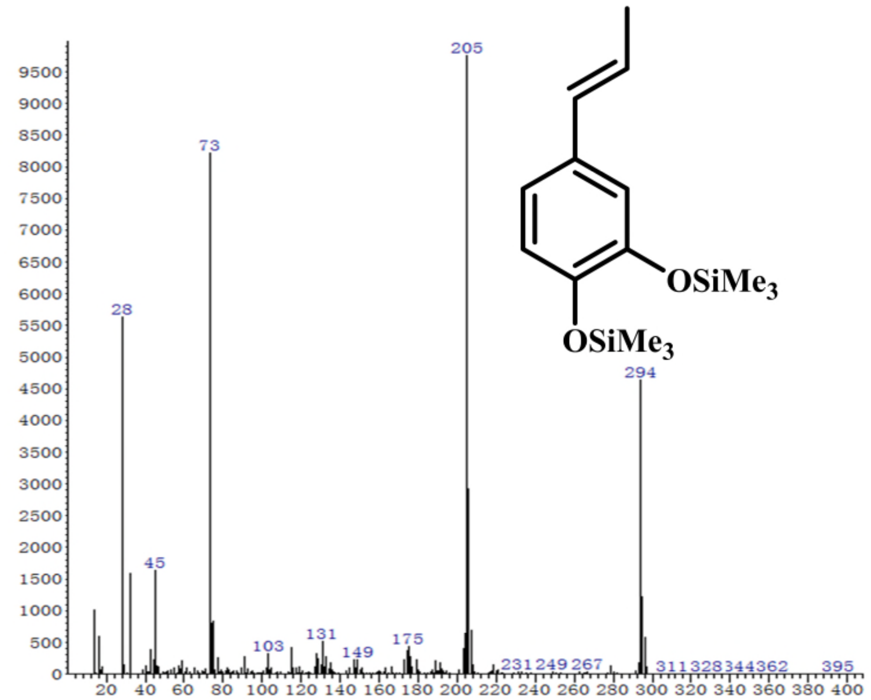

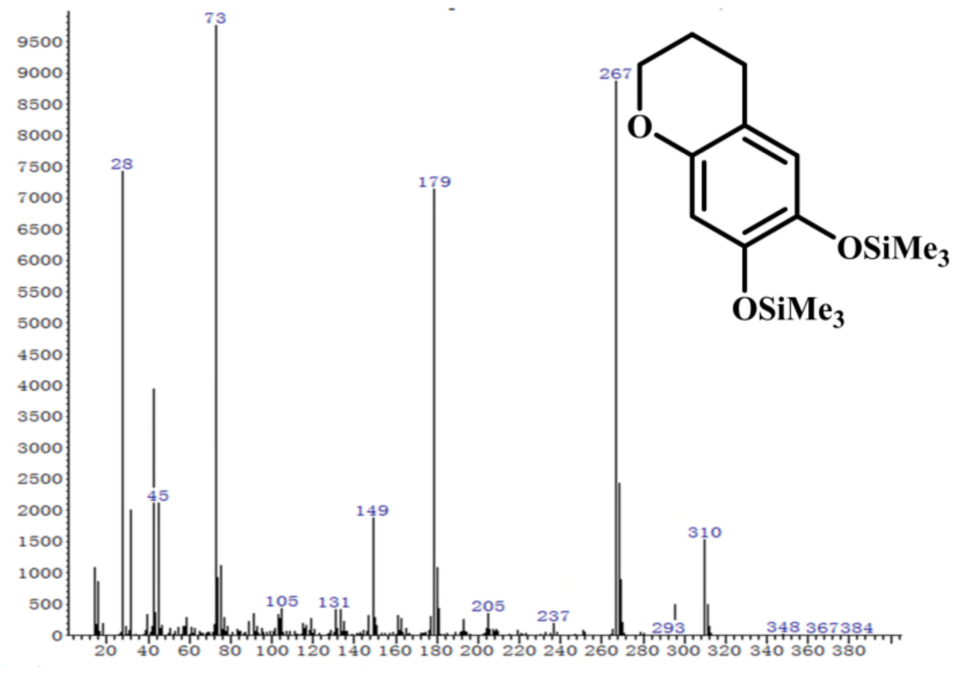

Supplementary Fig. 7 Mass spectra of product from C-lignin depolymerisation

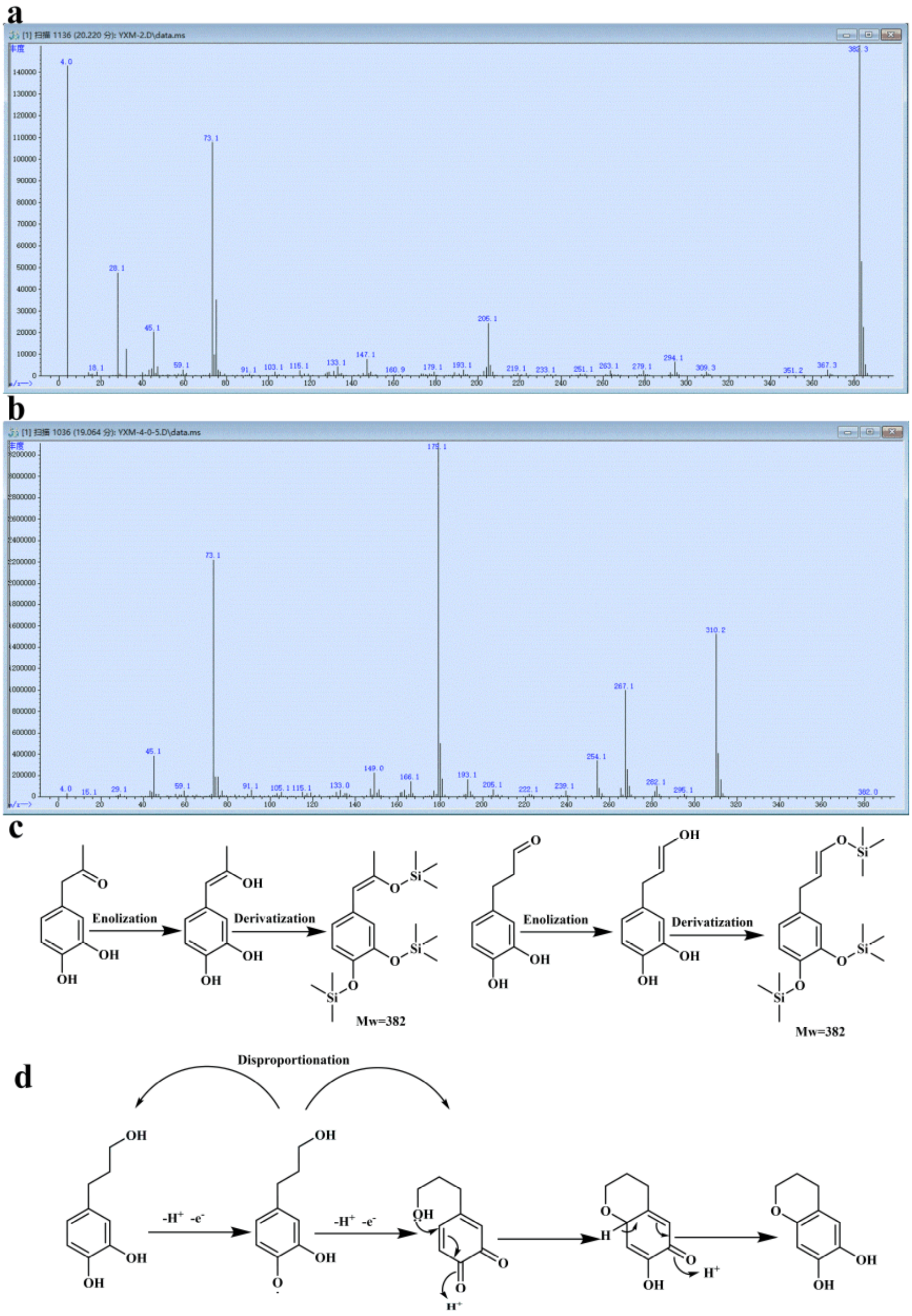

Supplementary Fig. 8 The MS spectrum of (a) 1-(3,4-dihydroxyphenyl)propan-2-one and (b) 3-(3,4-dihydroxyphenyl)propanal after BSFTA derivatization, (c) The derivatization experiments of 1-(3,4-dihydroxyphenyl)propan-2-one nor 3-(3,4-dihydroxyphenyl)propanal, and (d) The potential pathway to C5 formation

Supplementary Note 3. Structural Validation of C5 Product

To further clarify the structural identity of the C5 product obtained from C-lignin depolymerization, additional experiments and mechanistic analysis were conducted. In this study, BSTFA derivatization was employed to reduce the boiling points of diphenolic compounds, facilitating their detection and quantification by GC. To test the hypothesis that C5 might be 1-(3,4-dihydroxyphenyl)propan-2-one or 3-(3,4-dihydroxyphenyl)propanal, both commercial compounds were obtained and structurally verified by NMR spectroscopy. Subsequent BSTFA derivatization experiments were conducted in pyridine. The results indicated that both compounds undergo keto-enol tautomerization, wherein the enol form contains a reactive hydroxyl group that rapidly reacts with BSTFA, thereby disrupting the equilibrium (Supplementary Fig. 8c). After derivatization, all three active hydroxyl groups in each compound were fully silylated, and GC–MS analysis showed a molecular ion peak at m/z = 382 (Supplementary Fig. 8a and Fig 8b). In contrast, GC–MS analysis of the actual C5 product from C-lignin depolymerization revealed a molecular ion peak at m/z = 310, significantly differing from the above standards. These findings indicate that C5 is not 1-(3,4-dihydroxyphenyl)propan-2-one or 3-(3,4-dihydroxyphenyl)propanal. Instead, it is consistent with the proposed structure of Chroman-6,7-diol.

Chroman-6,7-diol is not commercially available, and its synthesis is nontrivial. Although we attempted to isolate this compound from the reaction mixture, its extremely low yield prevented acquisition of sufficient material for NMR verification. However, chroman-6,7-diol has been reported as a product of radical disproportionation from 4-(3-hydroxypropyl)-catechol (Supplementary Fig. 8d)[S12]. Notably, the GC–MS spectrum of our C5 compound closely matches that reported in the aforementioned study, further supporting our assignment.

Taken together, these experimental results and literature comparisons strongly support the identification of C5 as Chroman-6,7-diol, rather than any linear diphenolic ketone or aldehyde isomer.

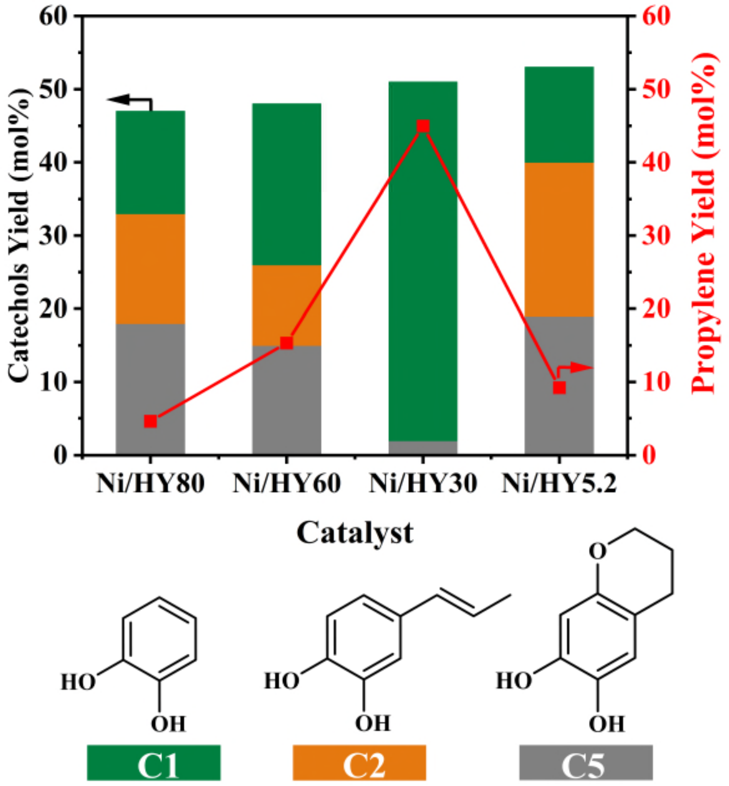

Supplementary Fig. 9 The effect of Si/Al ratio in support of catalyst on the monomer yields. Reaction condition: 50 mg C-lignin, 100 mg catalyst, 25 μL dodecane, 5 mL methanol/water mixture (1:4), 2 MPa H_2, 200 ^oC,12 h.

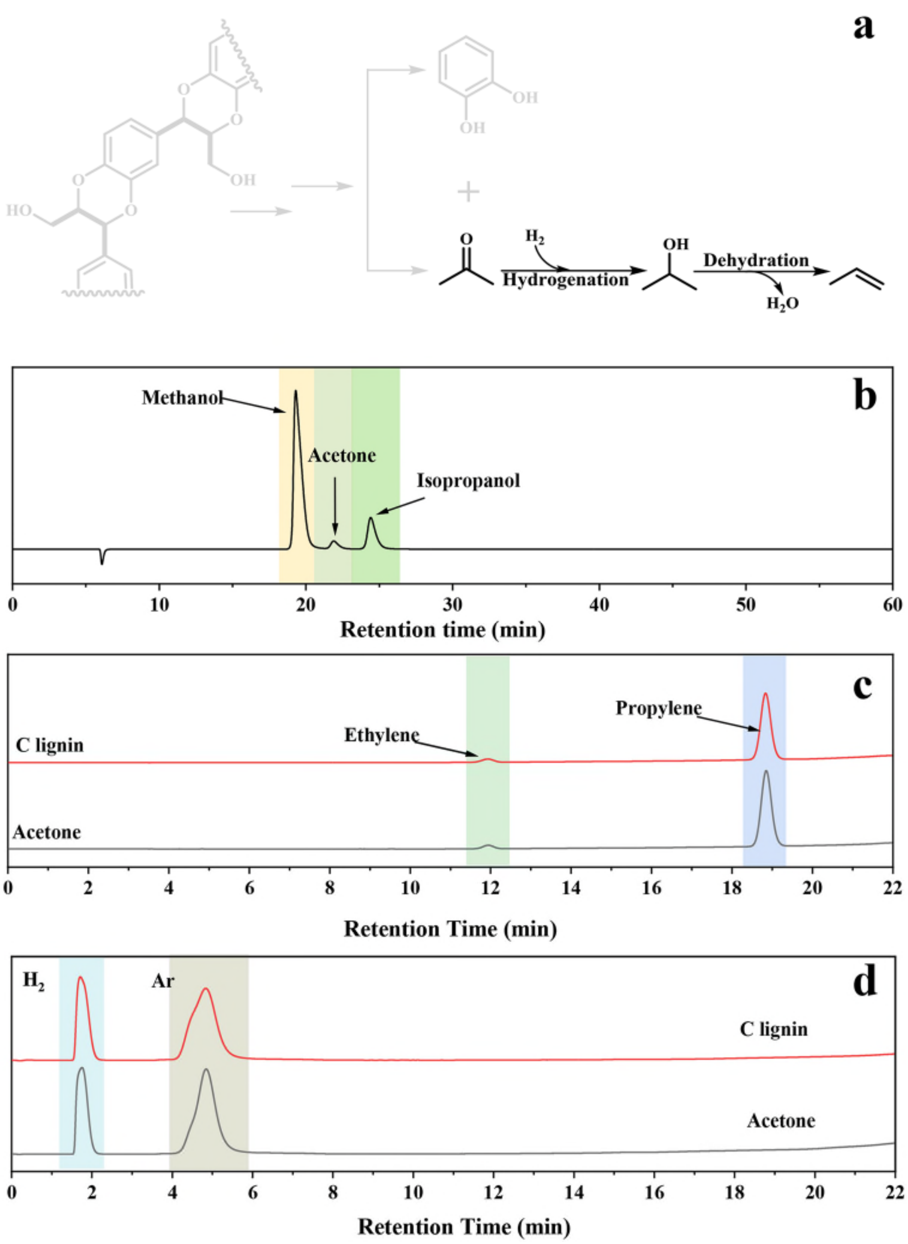

Supplementary Fig. 10 Conversion of acetone into propylene. (a) the pathway of acetone into propylene via hydrodehydration; (b) the HPLC spectra of the reaction mixture using acetone as raw material; (c) the GC spectra of the gaseous product in the reaction mixture using the FID detector; (d) the GC spectra of gaseous product in reaction mixture using TCD detector. Reaction condition: 100 μL acetone, 50 mg 0.97 wt% Ni/HY_{30}, 5 mL methanol/water mixture (1:4), 3 MPa H_2, 12 h.

Supplementary Table 13 The ICP-OES analysis of Ni/HY_{30} before and after the reaction and liquid phase

ICP-AES analysis (wt%)	Fresh Ni/HY_{30}	Spent Ni/HY_{30}[a]	Liquid phase[a]
Ni	0.97	0.91	Not detected

[a] reaction condition: C-lignin (50 mg), Ni/HY_{30} (100 mg), 25 μL dodecane, 5 mL methanol/water mixture (1:4), 200 °C, 3 MPa H_2, and 12 h.

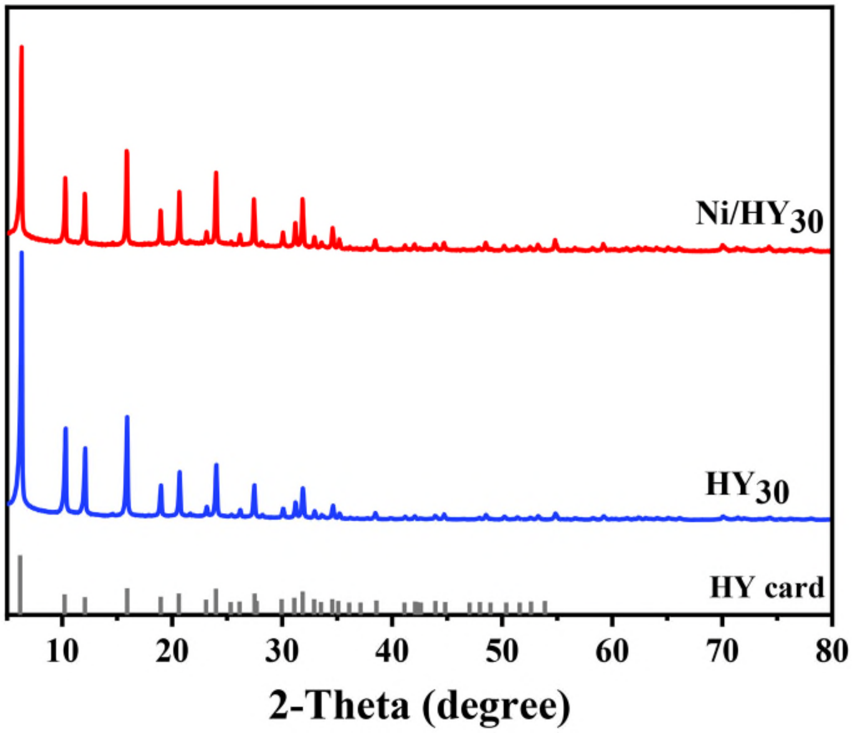

Supplementary Fig. 11 XRD patterns of HY_{30} and Ni/HY_{30} catalysts, a standard pattern of HY zeolite, are shown at the bottom.

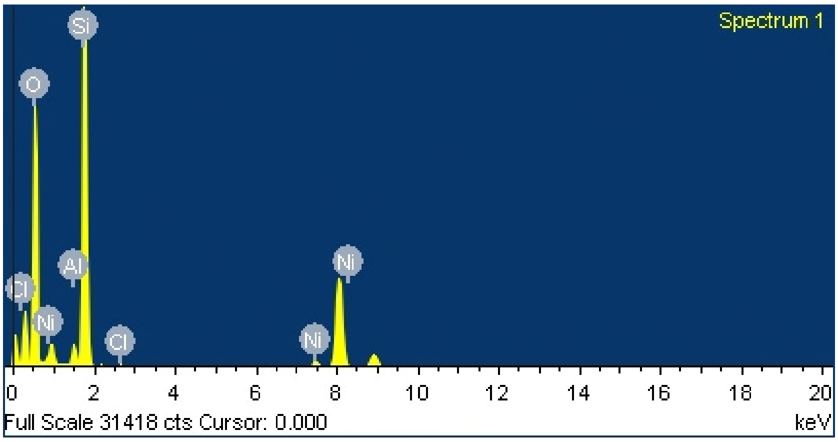

Supplementary Fig. 12 EDS images of 0.97 wt% Ni/HY_{30}

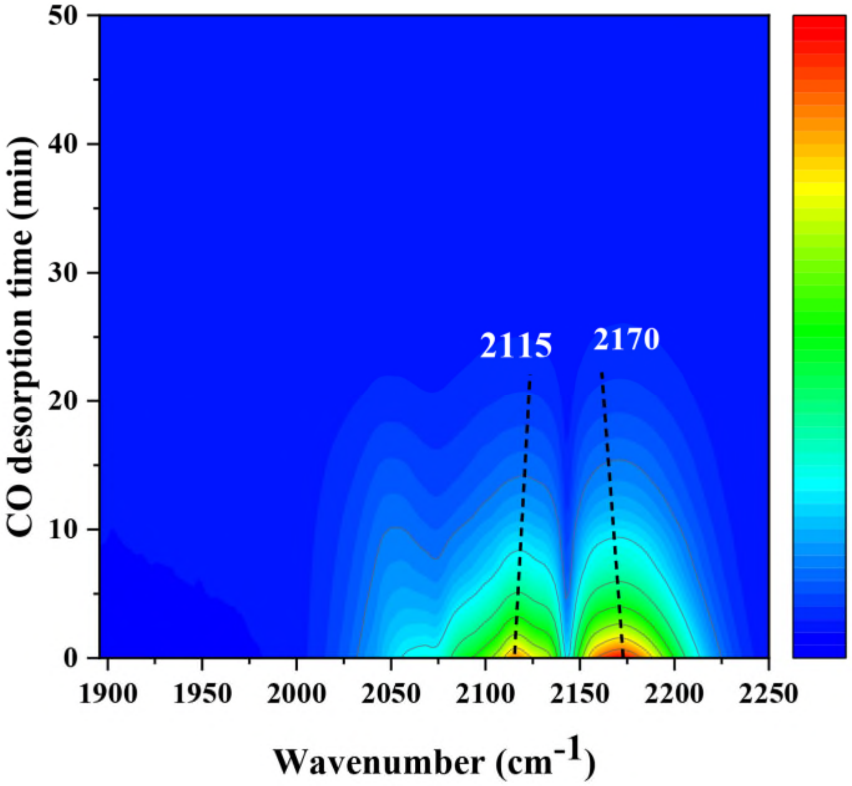

Supplementary Fig. 13 CO-FTIR spectra of HY_{30} at different desorption time

Supplementary Table 14 Dealkylation of propenylcatechol into catechol using different catalysts [a]

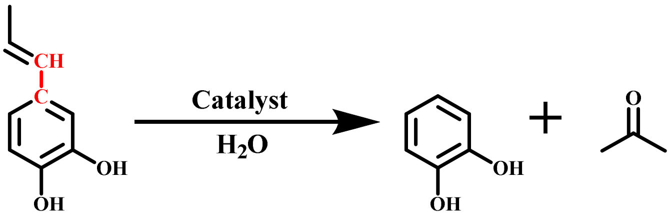

Entry	Catalyst	Yield (%)	
		Catechol	Acetone
1	HY_{30}	>99.0	51.4
2	None [b]	0	0
3	$HY_{5.2}$, HY_{60}, HY_{80},	5.5, 49.2, 30.1	0.6, 21.9, 15.4
4	$ZSM\text{-}5_{25}$, $ZSM\text{-}5_{50}$, $ZSM\text{-}5_{100}$, $ZSM\text{-}5_{200}$	0, 0, 0, 19.8	0, 0, 0, 2.6
5	MOR_{2}, MOR_{12}, MOR_{34}	0, 0, 0	0, 0, 0
6	$Beta_{2.5}$，$Beta_{25}$，$Beta_{40}$	0, 0, 0	0, 0, 0
7	MCM-22, MCM-41	0, 0	0, 0
8	SAPO-34	2.5	0.1
9	HCl (37 %) [c]	25.2	10.2
10	HY_{30} [d]	50.3	39.3
11	HY_{30} [e]	5.2	0.5

[a] Reaction condition： 100 mg substrate, 100 mg HY_{30} catalyst, 5 ml H_2O, 0.1 MPa Ar, 180 ^{o}C, 12 h; [b] without a catalyst; [c] 20 mg; [d] CH_3OH instead of H_2O; [e] CH_3CH_2OH instead of H_2O.

Supplementary Table 15 Textural properties of HY zeolite

**Texture properties**	**HY_{30}**	**HY_{30}-0.4**	**HY_{30}-0.8**	**HY_{30}-1.6**	**HY_{30}-2.4**	**HY_{30}-C**
Micropore Surface Area ($m^2 \cdot g^{-1}$)	301.05	283.46	271.28	254.43	247.82	245.18
Mesopore Surface Area ($m^2 \cdot g^{-1}$)	213.71	242.32	254.35	270.10	277.09	87.55
Micropore Volume ($cm^3 \cdot g^{-1}$)	0.201	0.190	0.176	0.156	0.151	0.134
Mesopore Volume ($cm^3 \cdot g^{-1}$)	0.188	0.180	0.205	0.214	0.520	0.087

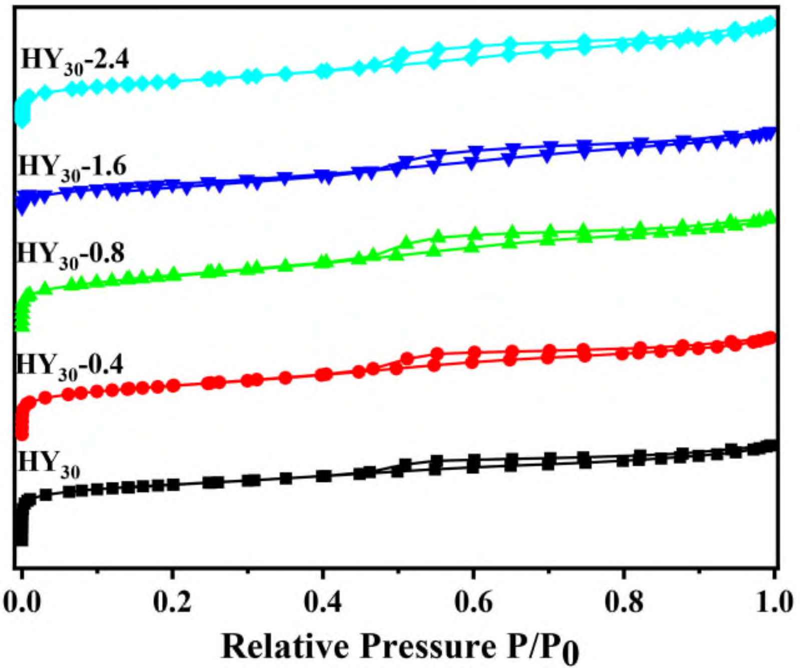

Supplementary Fig. 14 Nitrogen adsorption-desorption isotherm of HY_{30}, HY_{30}-0.4, HY_{30}-0.8, HY_{30}-1.6 and HY_{30}-2.4zeolite

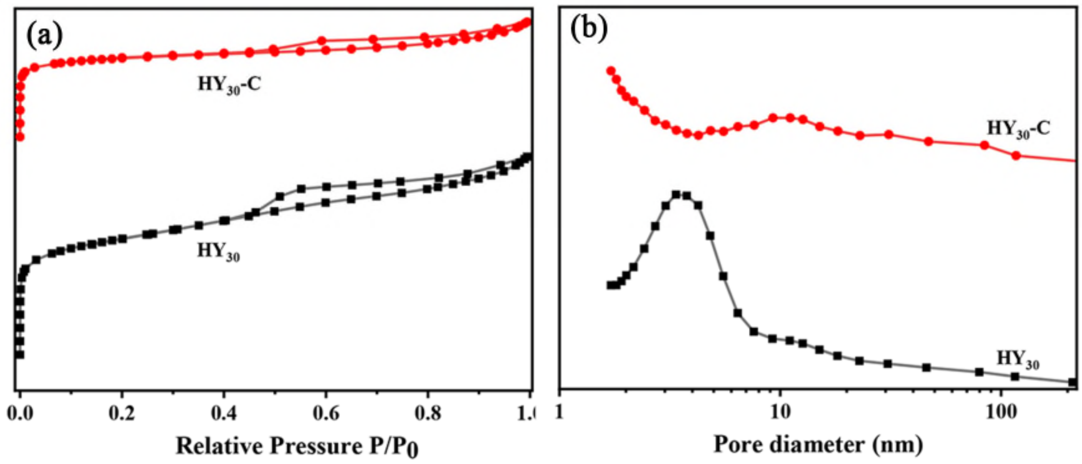

Supplementary Fig. 15 (a) Nitrogen adsorption-desorption isotherm of HY_{30} and HY_{30}-C; (b) pore size distribution of HY_{30} and HY_{30}-C

Supplementary Table 16 The chemical shift value (δ, ppm) of ^{13}C NMR spectrum of the intermediates in the reaction of C2 compound

ppm	Assignment
12.4	$C_{\gamma 2}$ in C7 compound
16.8	$C_{\gamma 1}$ in C2 compound
21.9	$C_{\beta 3}$ in C8 compound
29.4	$C_{\beta 1}$ in C7 compound
36.7	$C_{\beta 2}$ in C8 compound
42.0	$C_{\alpha 2}$ in C8 compound
46.9	$C_{\alpha 1}$ in C7 compound
50.3<br>56.6	Aromatic carbocation in C9 compound

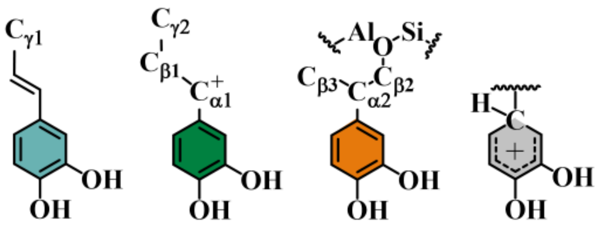

C2 C7 C8 C9

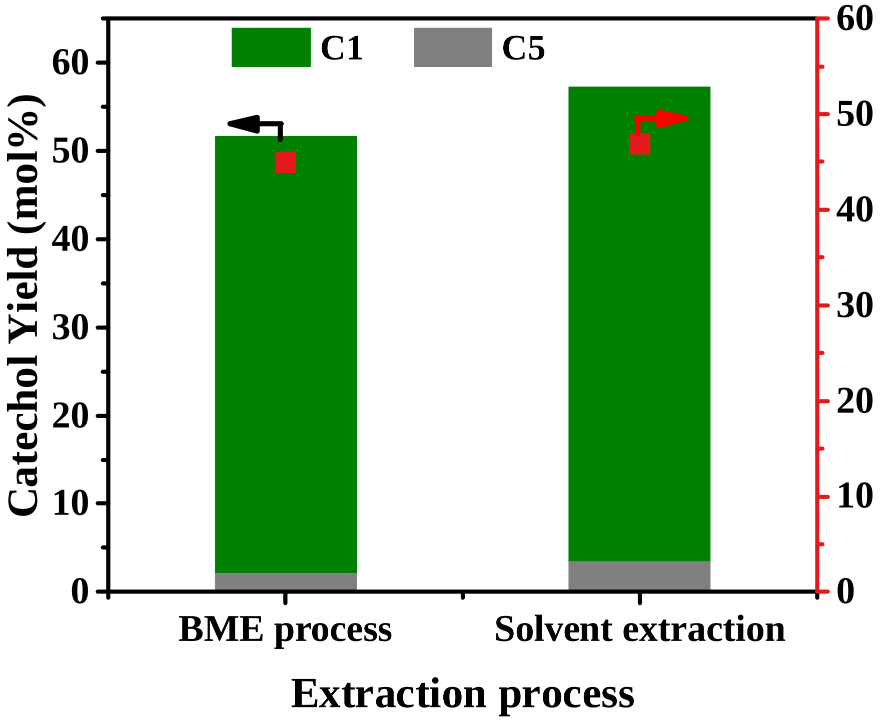

Supplementary Fig. 16 Hydrogenolysis-dealkylation of C-lignin obtained via BME process and solvent extraction over Ni/HY_{30} catalyst. Reaction condition: 50 mg C-lignin sample, 100 mg 0.97 wt% Ni/HY_{30}, 25 μL dodecane, 5 mL methanol/water mixture (1:4), 3 MPa H_2, 200 °C, 12 h

References

S1 Shen, X.-J., Chen, T., Wang, H.-M., Mei, Q., Yue, F., Sun, S., Wen, J.-L., Yuan, T.-Q., Sun, R.-C. Structural and morphological transformations of lignin macromolecules during bio-based deep eutectic solvent (DES) pretreatment. *ACS Sustain. Chem. Eng.* **8(5)**, 2130-2137. (2020)

S2 Kresse, G., Furthmüller, J. Efficiency of ab-initio total energy calculations for metals and semiconductors using a plane-wave basis set. *Comput. Mater. Sci.* **6**, 15−50 (1996)

S3 Kresse, G., Furthmüller, J. Efficient iterative schemes for ab initio total-energy calculations using a plane-wave basis set. *Phys. Rev. B* **54**, 11169−11186 (1996).

S4 Perdew, J., Burke, K., Ernzerhof, M. Generalised gradient approximation made simple. *Phys. Rev. Lett.* **77**, 3865−3868 (1996).

S5 Kresse, G., Joubert, D. From ultrasoft pseudopotentials to the projector augmented-wave method. *Phys. Rev. B* **59**, 1758-1775 (1999).

S6 Blöchl, P. Projector augmented-wave method. Projector augmented-wave method. *Phys. Rev. B* **50**, 17953−17979 (1994).

S7 Grimme, S., Antony, J., Ehrlich, S., Krieg, H. A consistent and accurate ab initio parametrisation of density functional dispersion correction (DFT-D) for the 94 elements H-Pu. *J. Chem. Phys.* **132**, 154104 (2010).

S8 Montazeri M, Eckelman M J. Life cycle assessment of catechols from lignin depolymerization[J]. ACS Sustainable Chemistry & Engineering, 2016, 4(3): 708-718.

S9 Catechol faces supply crunch while prices rise. https://www.gep.com/blog/mind/catechol-faces-supply-crunch-while-prices-rise (2018).

S10 Global catechol (CAS 120-80-9) market 2021 progress insight, CAGR 8.44% value, share, growth rate, business demand, industry outlook top manufacturers and forecast to 2027.

https://www.rfdtv.com/story/44044564/Global-Catechol-(CAS-120-80-9)-Market-2021-Progress-Insight-CAGR-8.44-Value-Share-Growth-Rate-Business-Demand-Industry-Outlook-Top-Manufacturers-and-Forecast-to-2027 (2021).

S11 Sluiter, A., Hames, B., Ruiz, R., Scarlata, C., Sluiter, J., Templeton, D., Crocker, D., 2008b. Determination of structural carbohydrates and lignin in biomass. NREL/TP-510-42618, Laboratory Analytical Procedure (LAPs). National Renewable Energy Laboratory, Golden CO

S12 Li, Y., Shuai, L., Kim, H., Motagamwala, A. H., Mobley, J. K., Yue, F., . Tobimatsu, Y., Havkin-Frenkel D., Chen F., Dixon R., Luterbacher J., Dumesic J., Ralph, J. (2018). An "ideal lignin" facilitates full biomass utilization. *Science Advances*, 4(9), eaau2968.

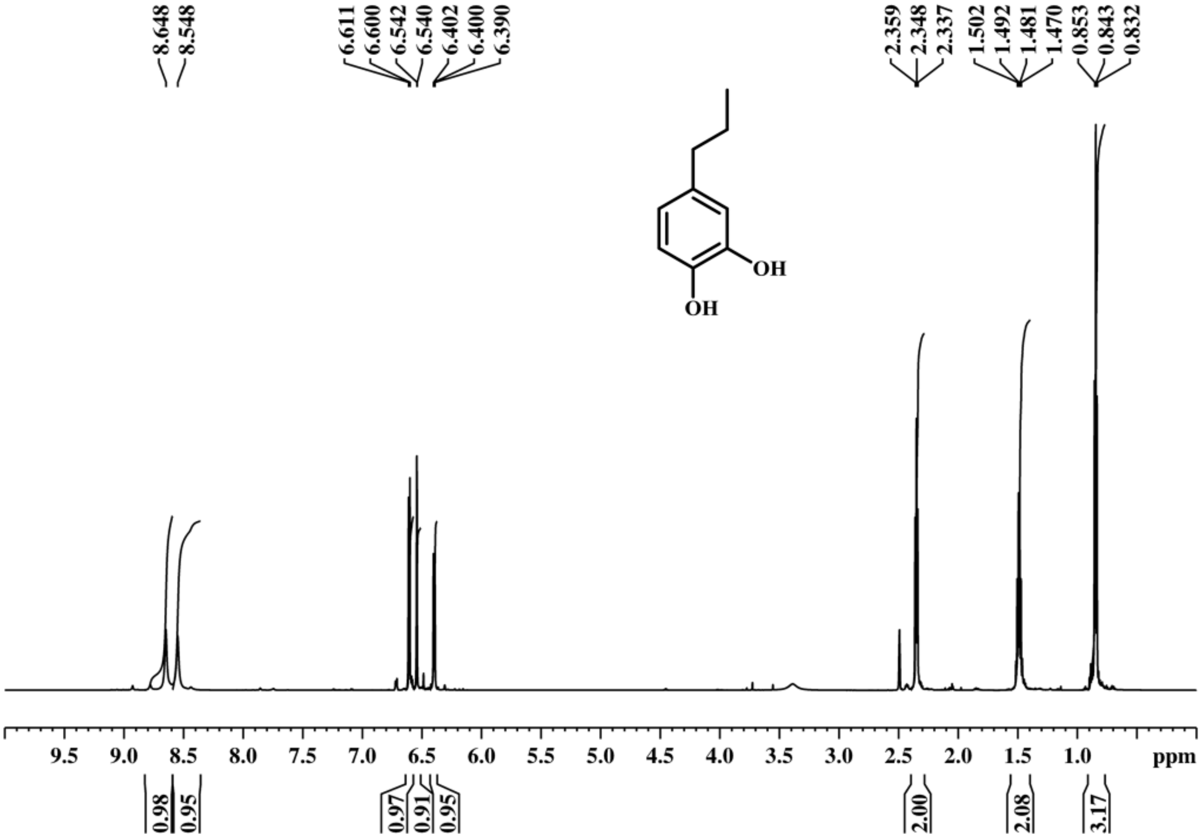

^{1}H-NMR of synthetic compound 4-propyl-catechol. Solvent: DMSO-*d6*.

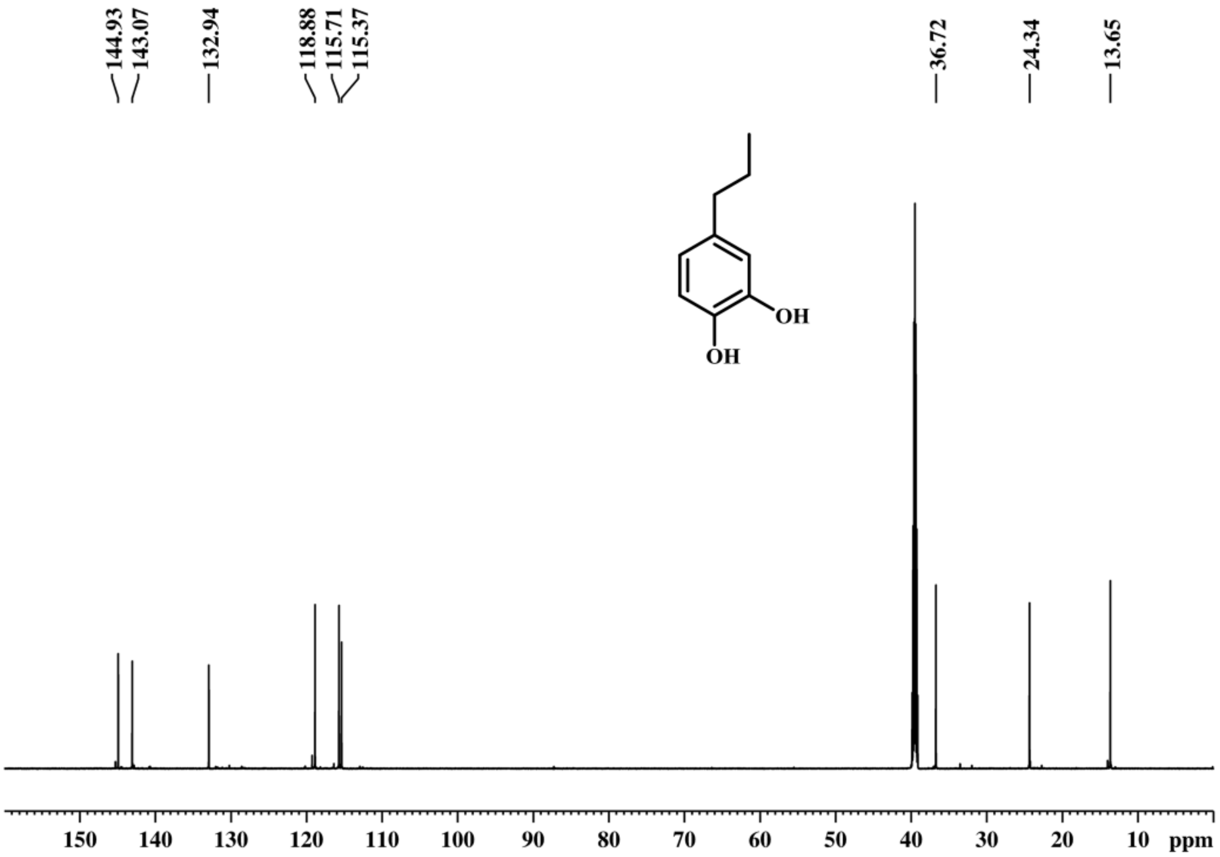

^{13}C-NMR of synthetic compound 4-propyl-catechol. Solvent: DMSO-*d6*.

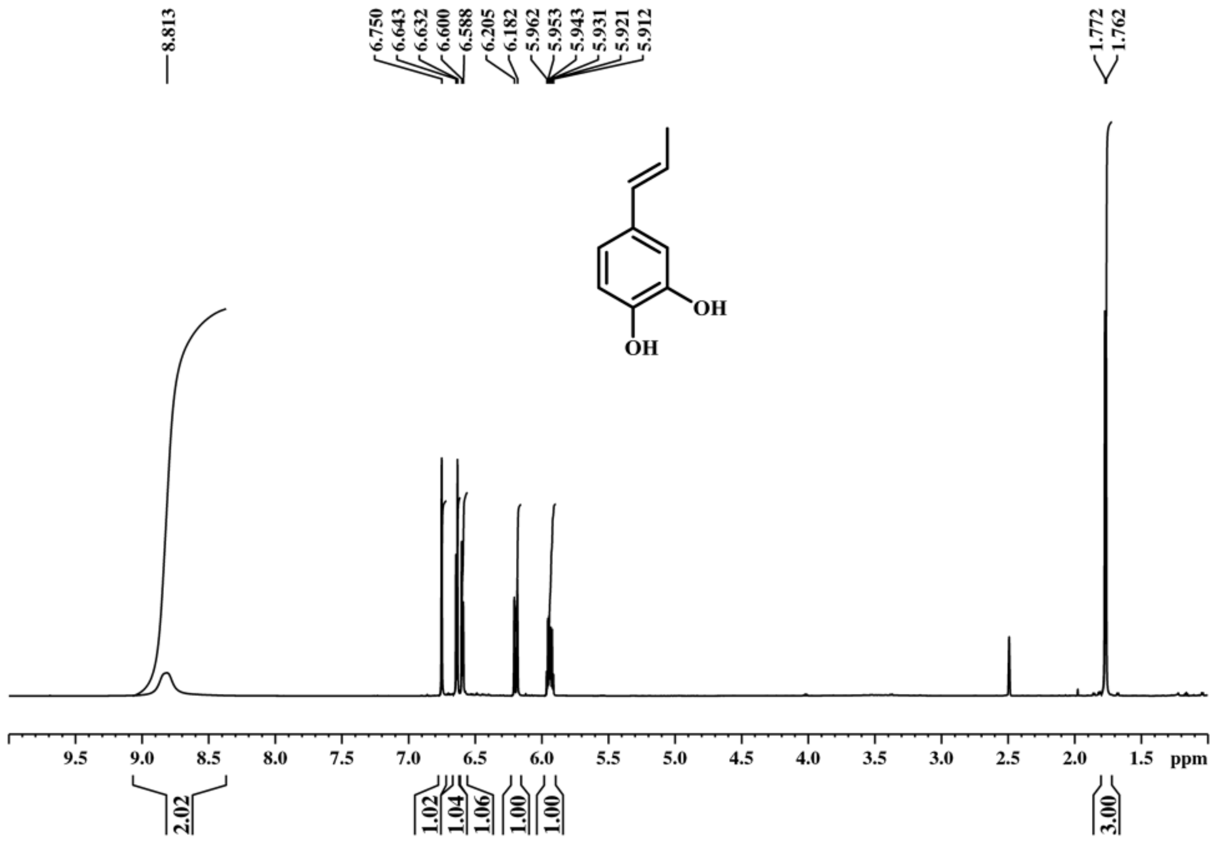

[1]**H-NMR of synthetic compound 4-propenyl-catechol**. Solvent: DMSO-*d6*.

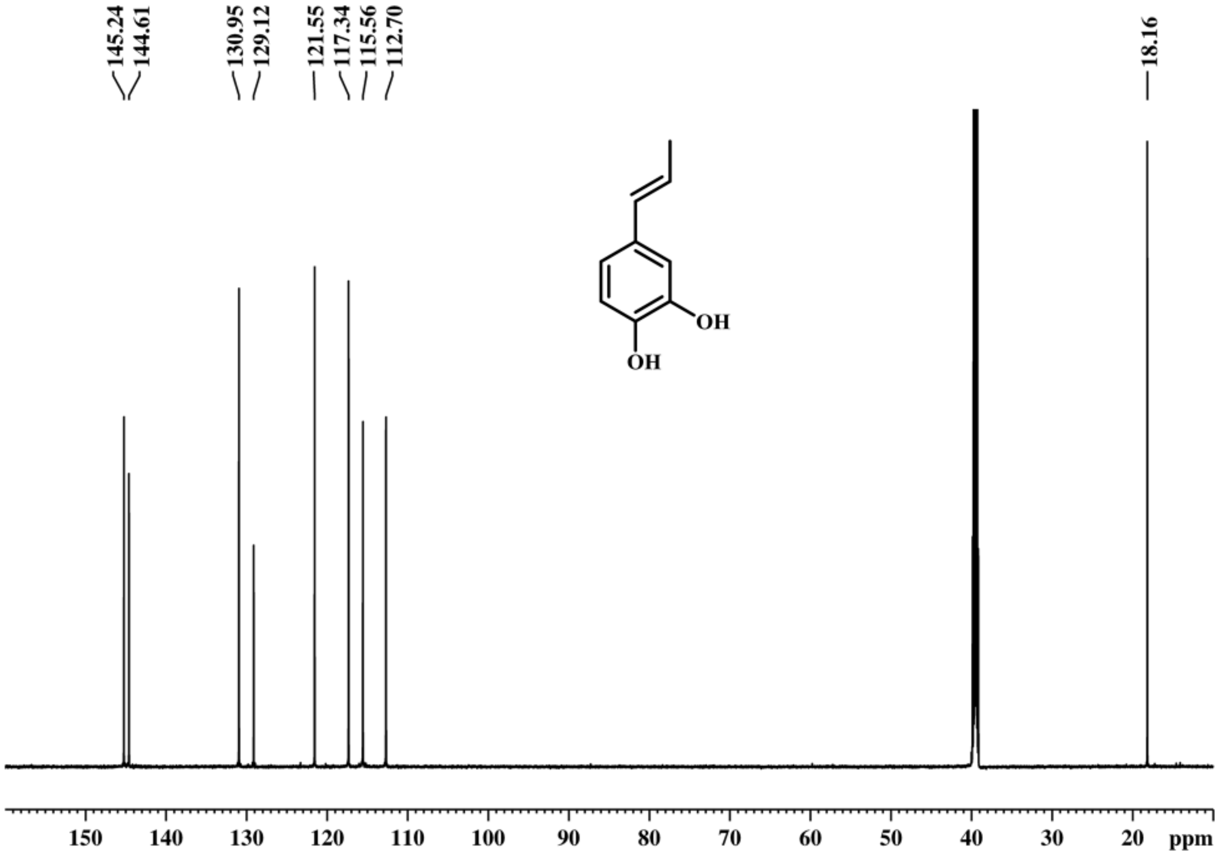

^{13}C-NMR of synthetic compound 4-propenyl-catechol. Solvent: DMSO-*d6*.

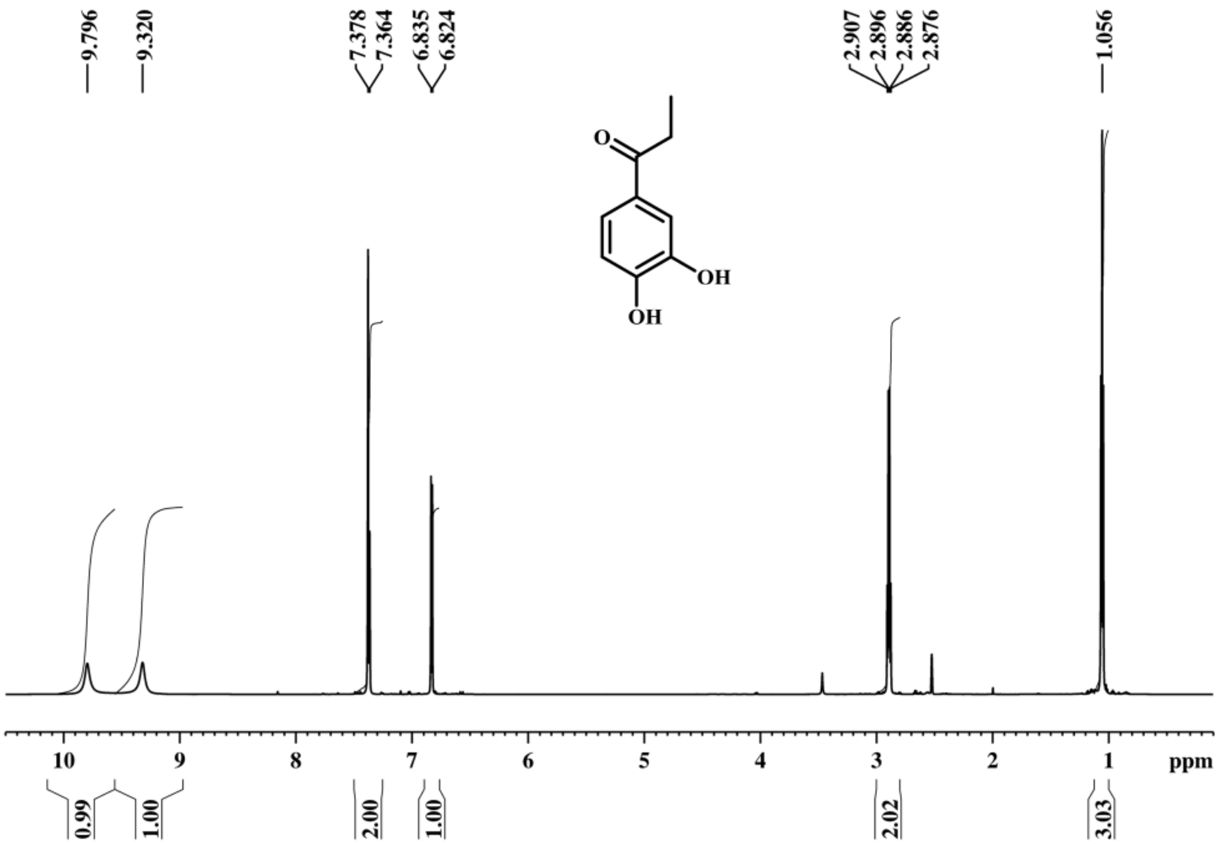

^{1}H-NMR of synthetic compound 3',4'-dihydroxypropiophenone. Solvent: DMSO-*d6*.

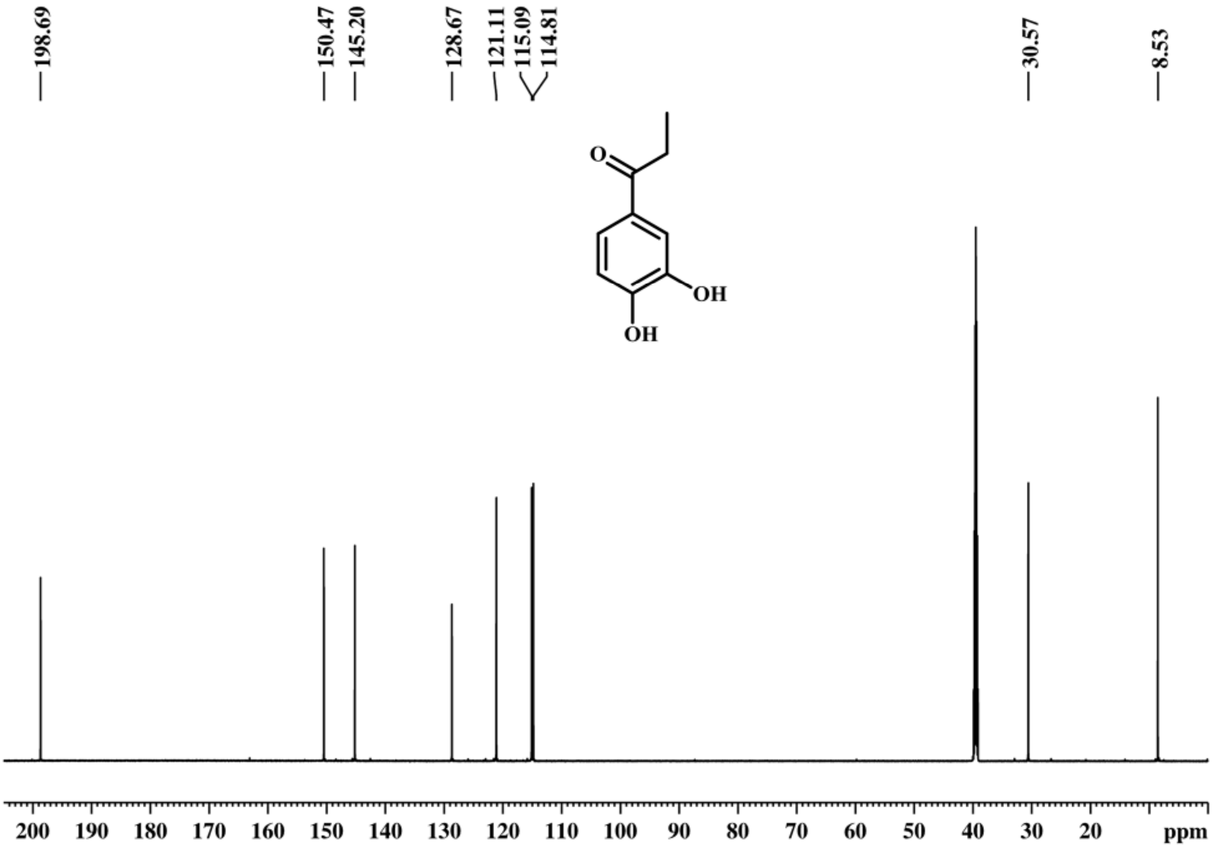

^{13}C-NMR of synthetic compound 3',4'-dihydroxypropiophenone. Solvent: DMSO-*d6*.

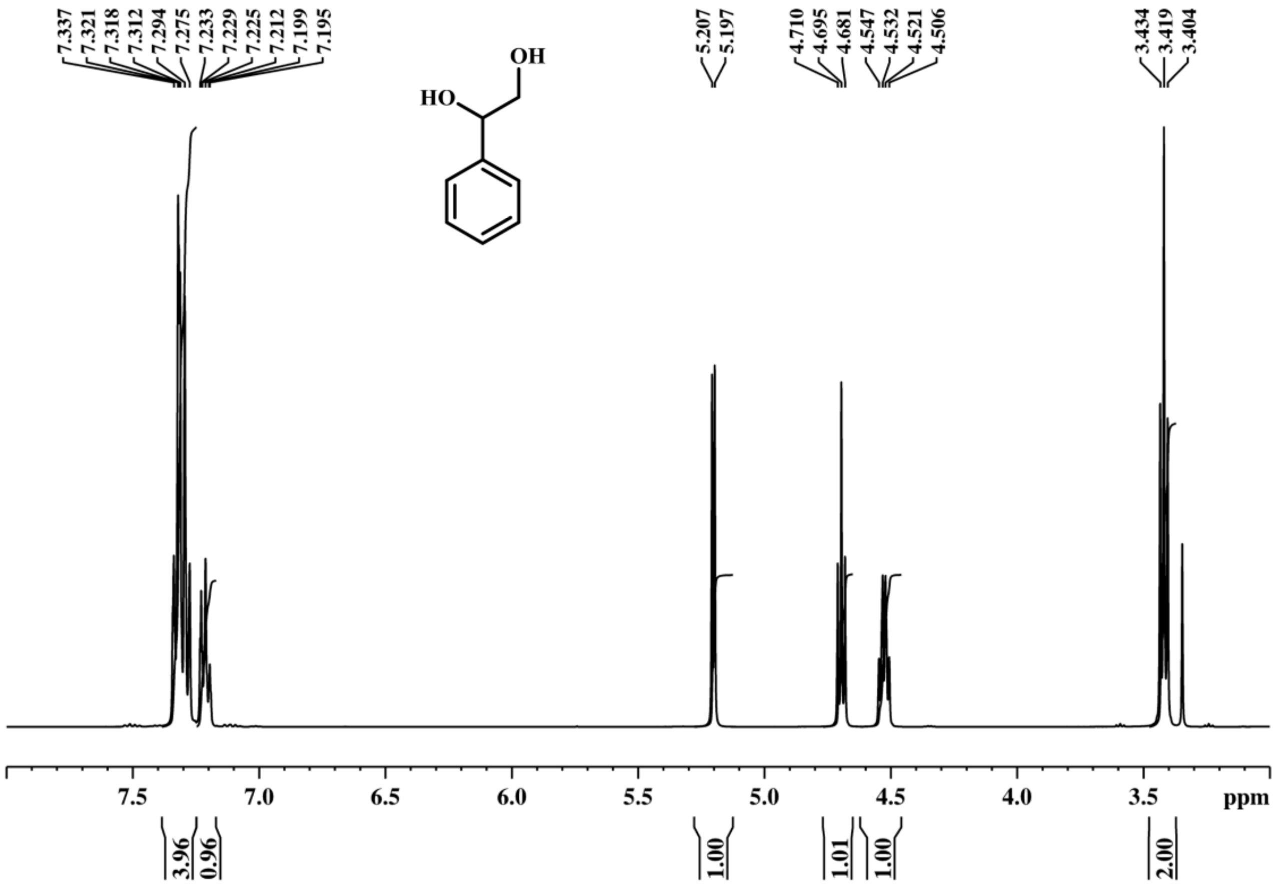

¹H-NMR of synthetic compound 1-phenylethane-1,2-diol. Solvent: DMSO-*d6*.

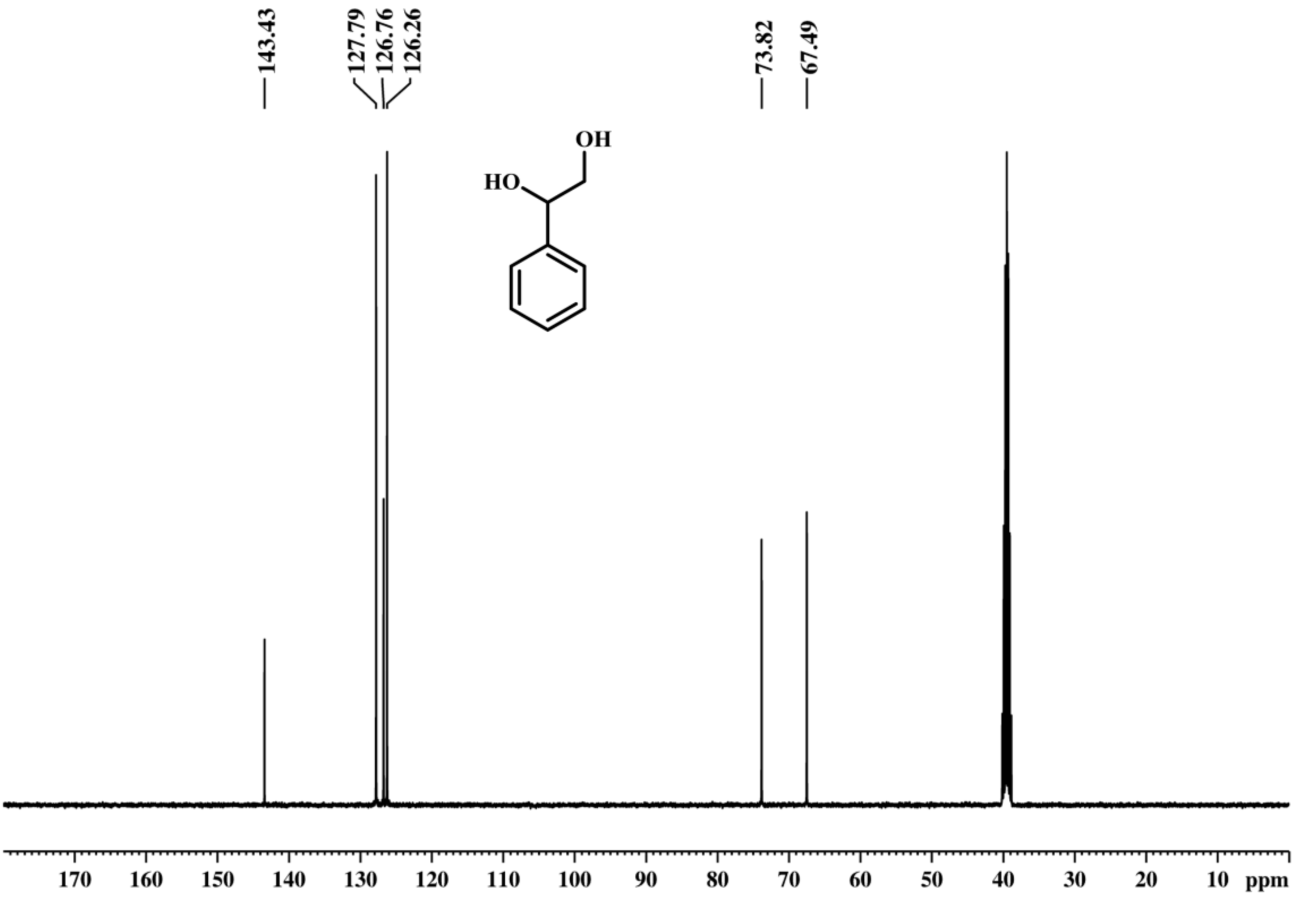

^{13}C-NMR of synthetic compound 1-phenylethane-1,2-diol. Solvent: DMSO-*d6*.

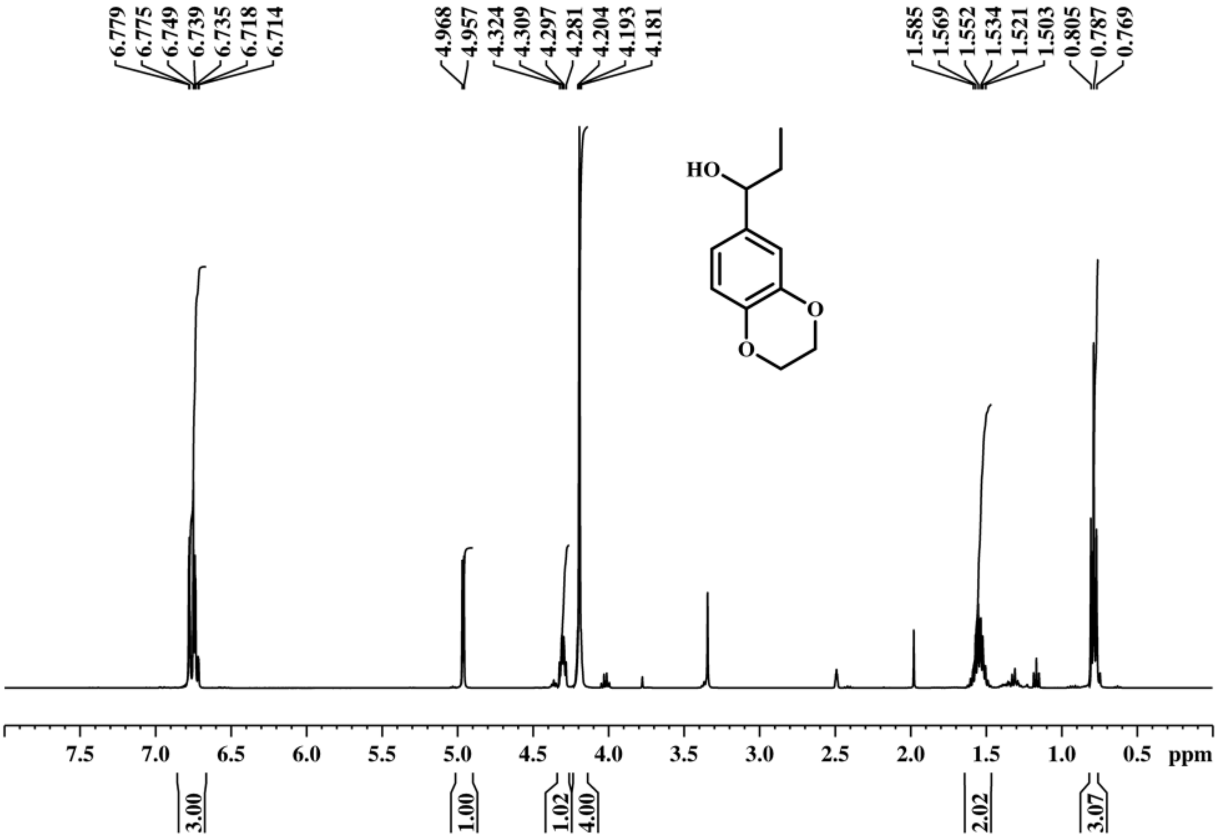

^{1}H-NMR of synthetic compound 1-(3,4-ethylenedioxyphenyl)propan-1-ol. Solvent: DMSO-*d6*.

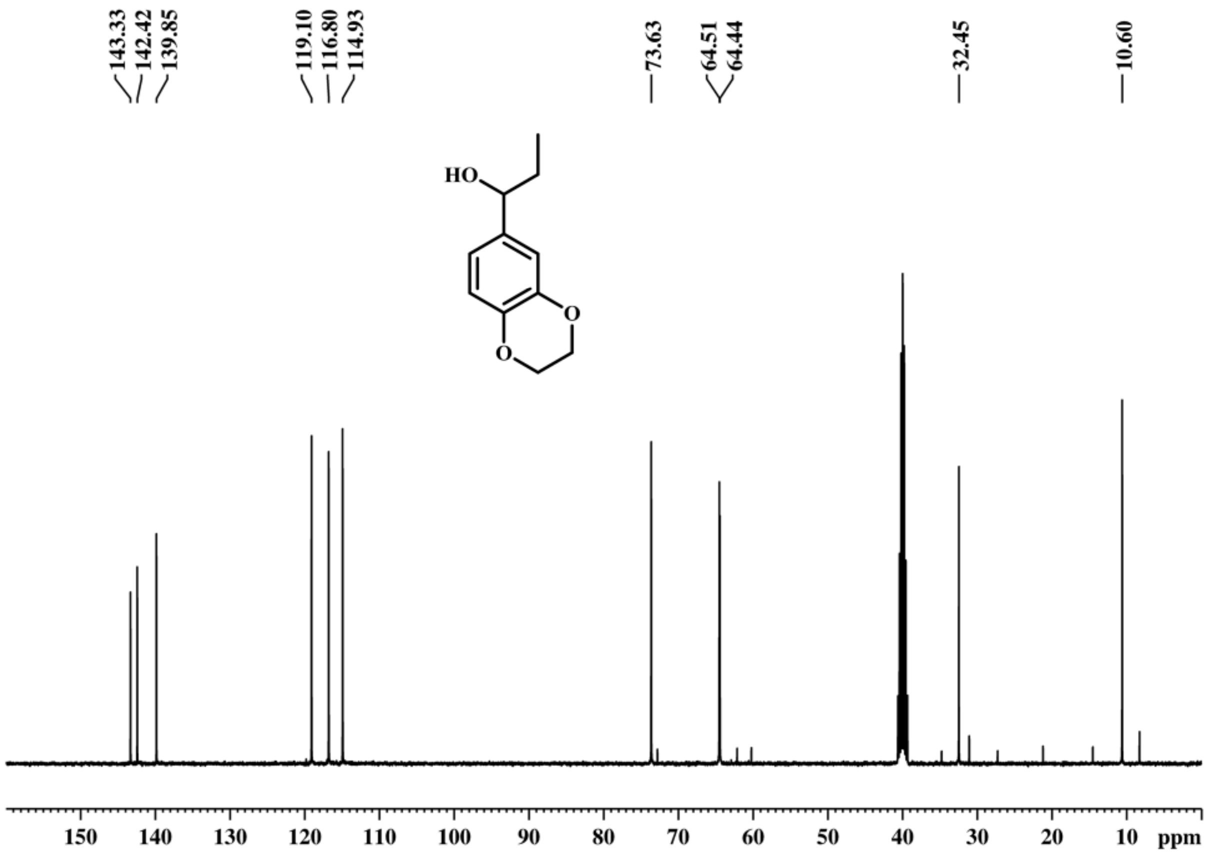

^{13}C-NMR of synthetic compound 1-(3,4-ethylenedioxyphenyl)propan-1-ol. Solvent: DMSO-*d6*.

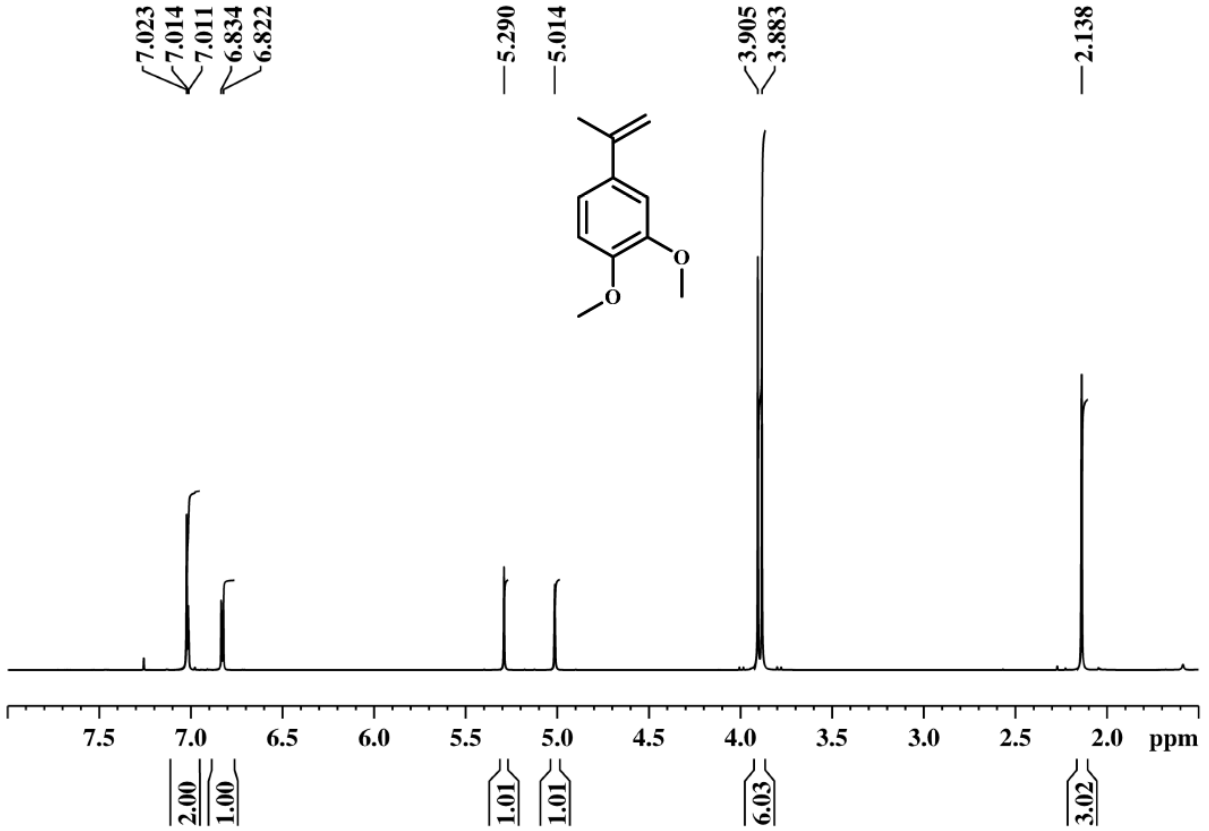

^{1}H-NMR of synthetic compound 4-isopropenyl-1,2-dimethoxybenzene. Solvent: DMSO-*d6*.

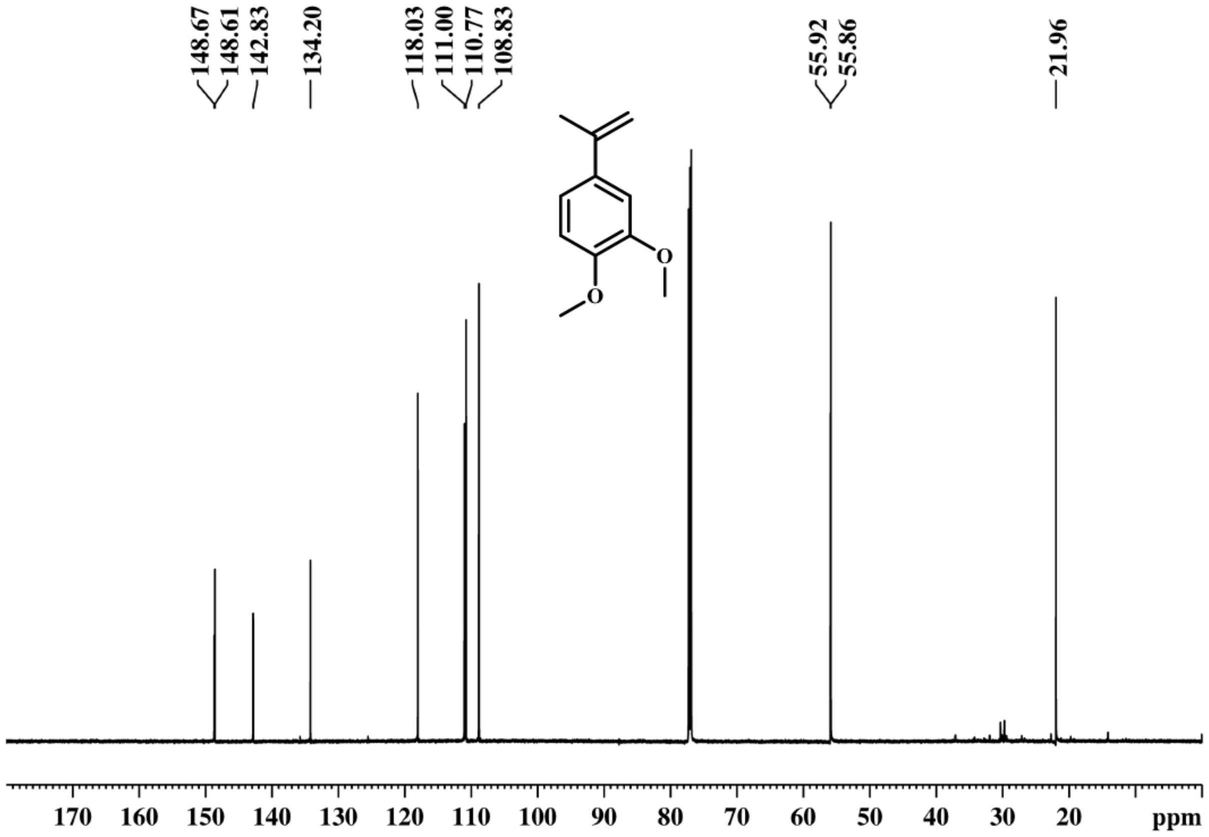

^{13}C-NMR of synthetic compound 4-isopropenyl-1,2-dimethoxybenzene. Solvent: DMSO-*d6*.

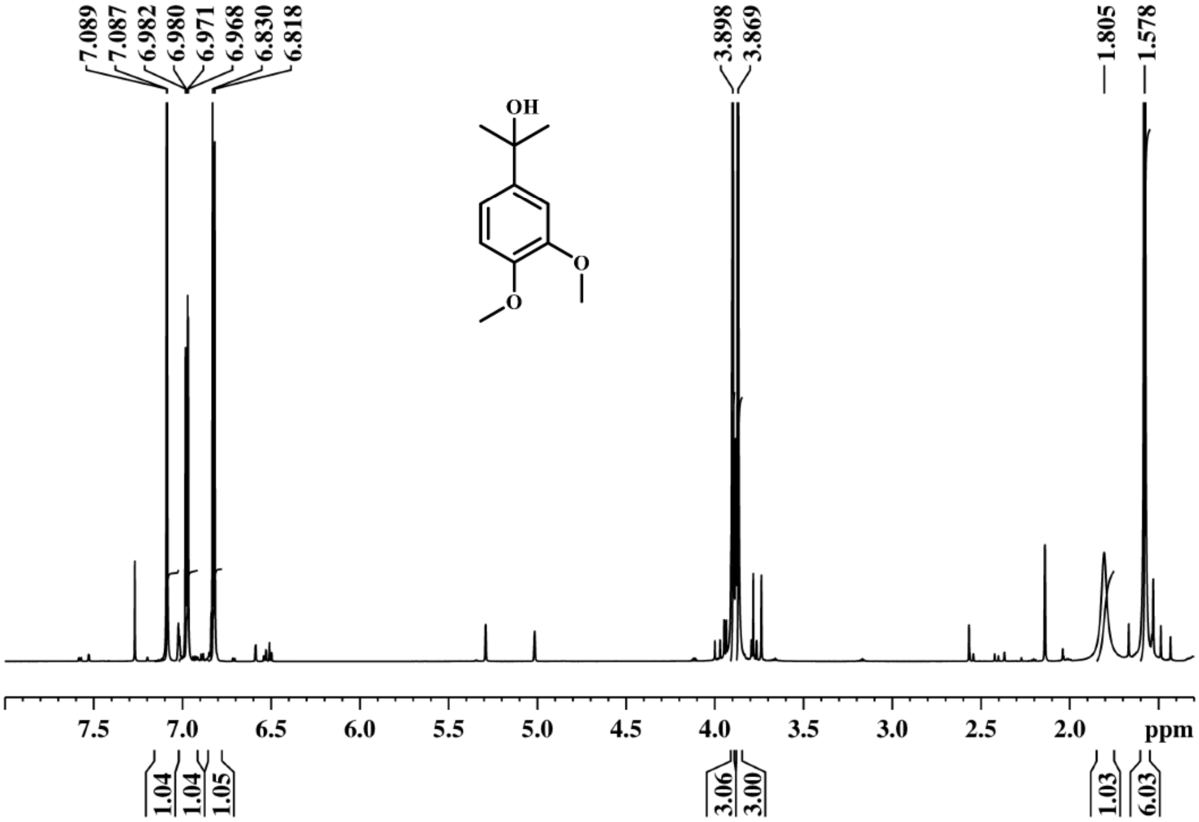

^{1}H-NMR of synthetic compound 3,4-dimethoxycumyl alcohol. Solvent: DMSO-*d6*.

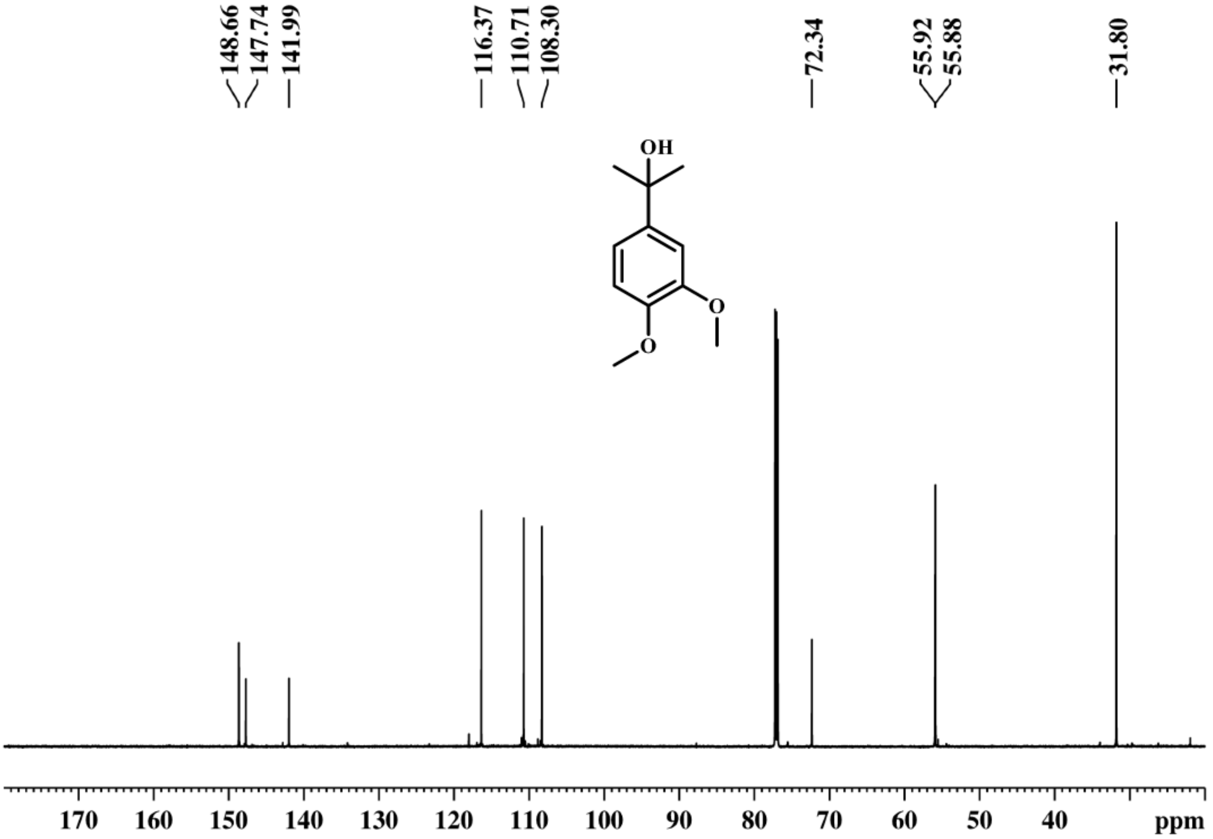

^{13}C-NMR of synthetic compound 3,4-dimethoxycumyl alcohol. Solvent: DMSO-*d6*.

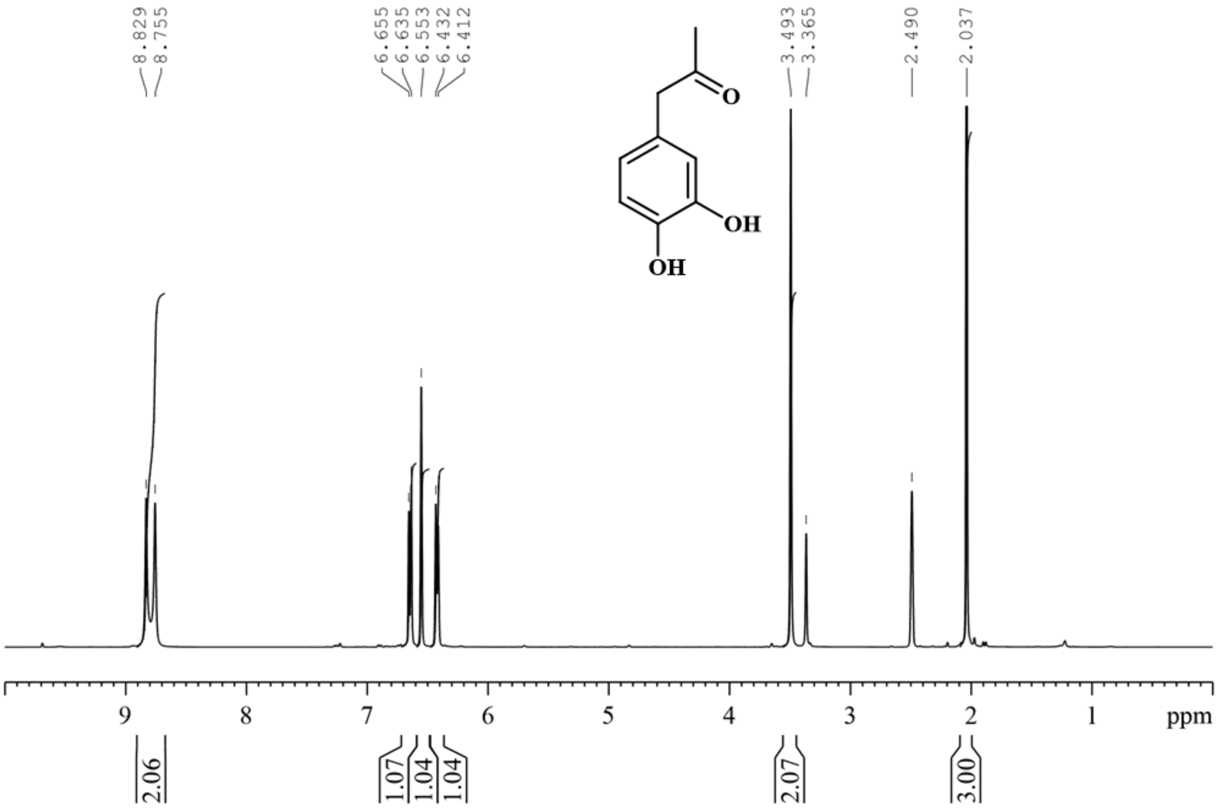

^{1}H-NMR of 1-(3,4-dihydroxyphenyl) propan-2-one. Solvent: DMSO-*d6*.

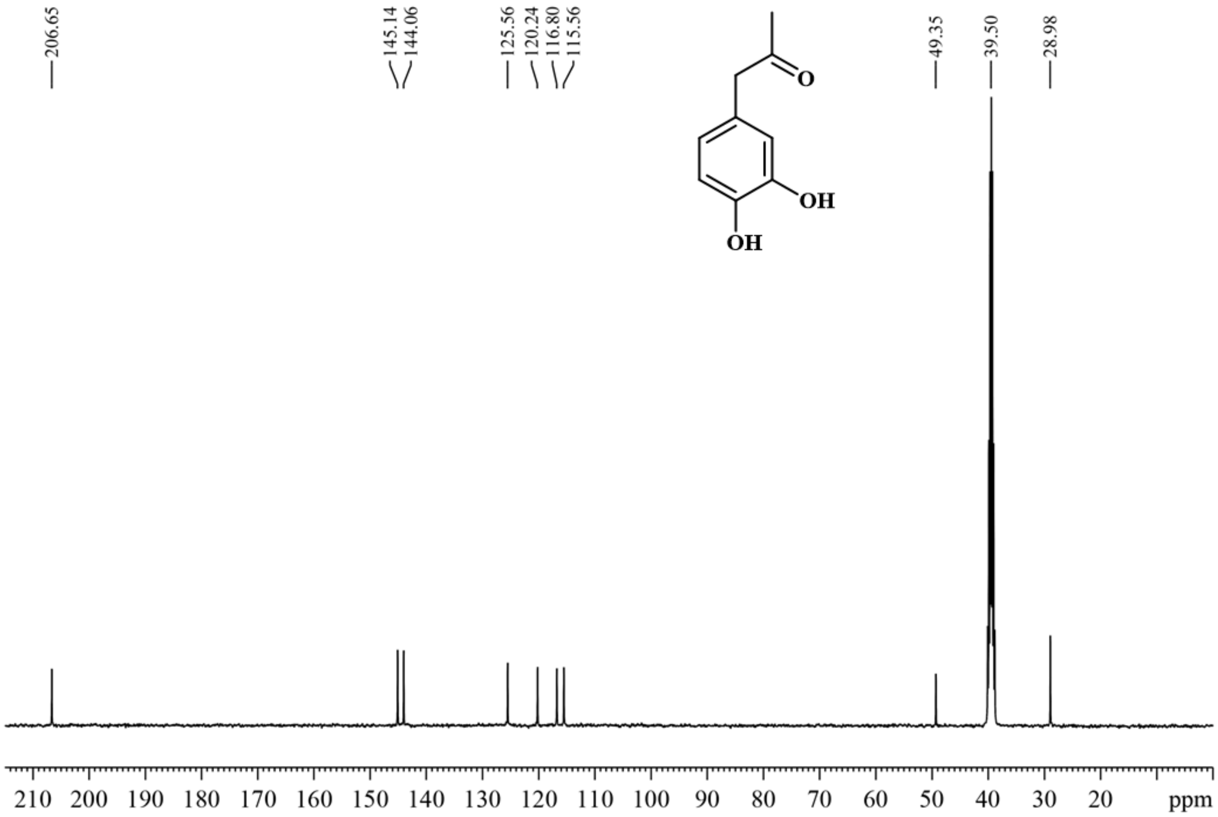

¹³C-NMR of 1-(3,4-dihydroxyphenyl) propan-2-one. Solvent: DMSO-*d6*.

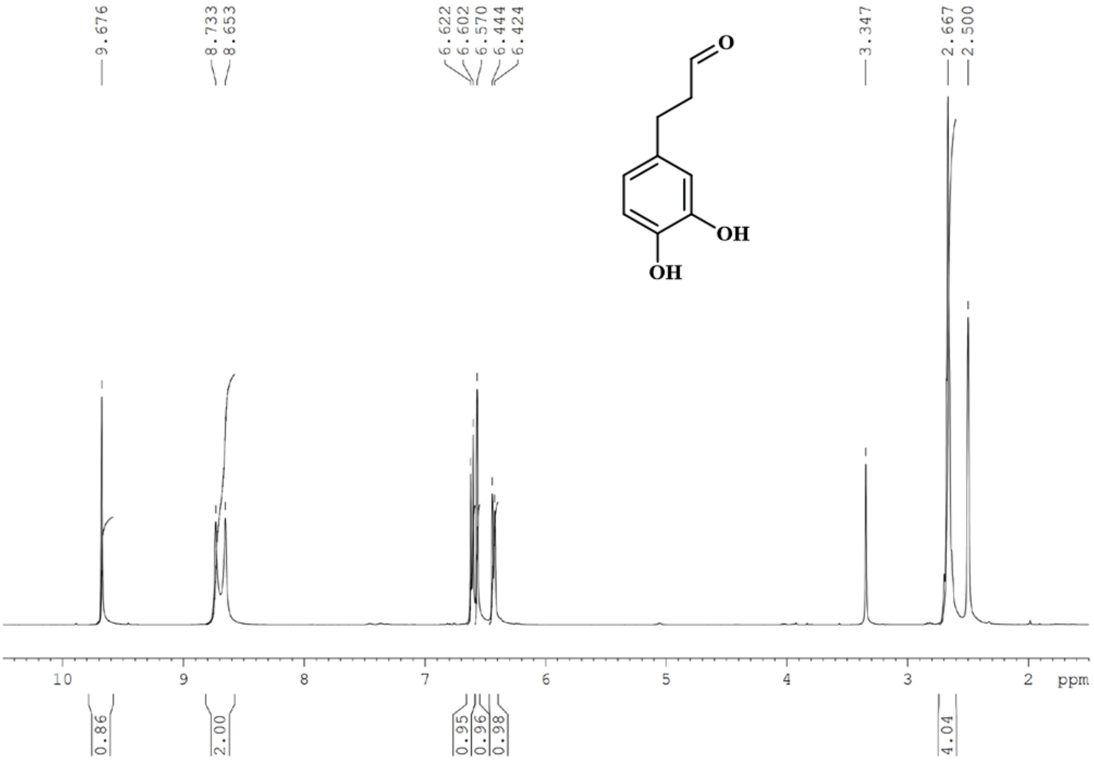

^{1}H-NMR of 3-(3,4-dihydroxyphenyl)propanal. Solvent: DMSO-*d6*.

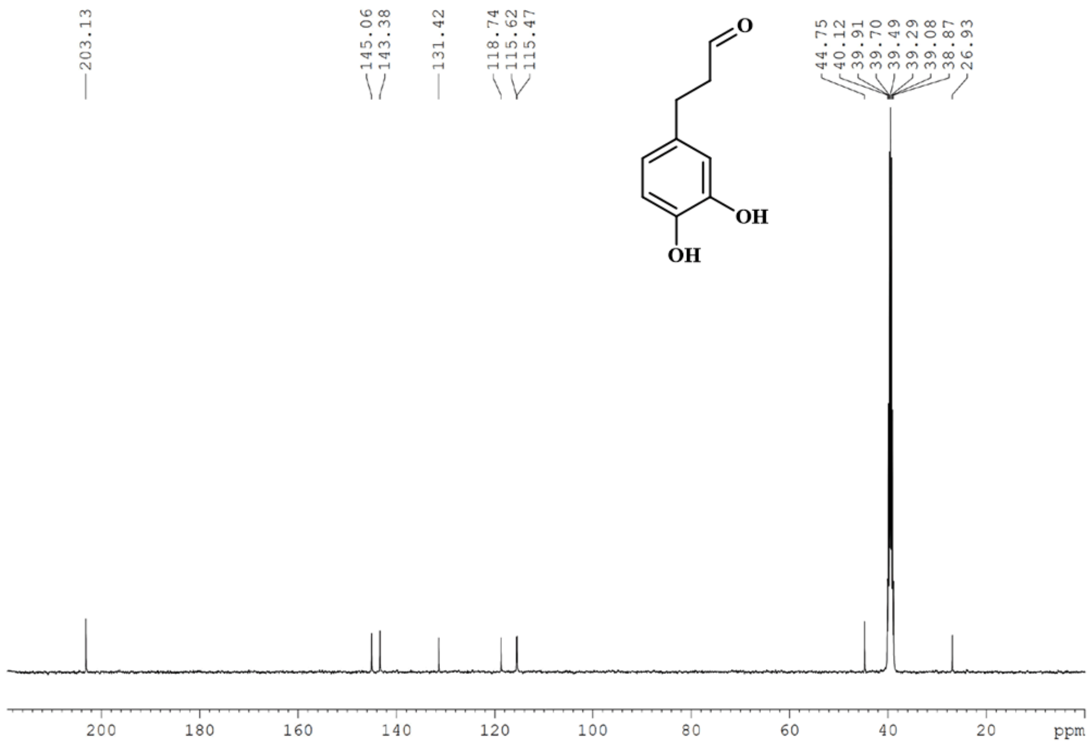

^{13}C-NMR of 3-(3,4-dihydroxyphenyl)propanal. Solvent: DMSO-*d6*.
